# Elongation of Long‐Chain Fatty Acid Family Member 6 (Elovl6)‐Driven Fatty Acid Metabolism Regulates Vascular Smooth Muscle Cell Phenotype Through AMP‐Activated Protein Kinase/Krüppel‐Like Factor 4 (AMPK/KLF4) Signaling

**DOI:** 10.1161/JAHA.116.004014

**Published:** 2016-11-23

**Authors:** Hiroaki Sunaga, Hiroki Matsui, Saki Anjo, Mas Risky A. A. Syamsunarno, Norimichi Koitabashi, Tatsuya Iso, Takashi Matsuzaka, Hitoshi Shimano, Tomoyuki Yokoyama, Masahiko Kurabayashi

**Affiliations:** ^1^ Department of Laboratory SciencesGunma University Graduate School of Health SciencesMaebashiJapan; ^2^ Department of Medicine and Biological SciencesGunma University Graduate School of MedicineMaebashiJapan; ^3^ Department of BiochemistryFaculty of Medicine Universitas PadjadjaranJatinangorIndonesia; ^4^ Department of Internal Medicine (Endocrinology and Metabolism)Faculty of MedicineUniversity of TsukubaTsukubaJapan; ^5^ Graduate School of Comprehensive Human Sciences International Institute for Integrative Sleep Medicine (WPI‐IIIS)TsukubaJapan

**Keywords:** Elovl6, fatty acid, neointimal hyperplasia, proliferation, smooth muscle cell, Animal Models of Human Disease, Lipids and Cholesterol, Smooth Muscle Proliferation and Differentiation, Vascular Biology

## Abstract

**Background:**

Fatty acids constitute the critical components of cell structure and function, and dysregulation of fatty acid composition may exert diverging vascular effects including proliferation, migration, and differentiation of vascular smooth muscle cells (VSMCs). However, direct evidence for this hypothesis has been lacking. We investigated the role of elongation of long‐chain fatty acid member 6 (Elovl6), a rate‐limiting enzyme catalyzing the elongation of saturated and monounsaturated long‐chain fatty acid, in the regulation of phenotypic switching of VSMC.

**Methods and Results:**

Neointima formation following wire injury was markedly inhibited in Elovl6‐null (Elovl6^−/−^) mice, and cultured VSMCs with siRNA‐mediated knockdown of Elovl6 was barely responsive to PDGF‐BB. Elovl6 inhibition induced cell cycle suppressors p53 and p21 and reduced the mammalian targets of rapamycin (mTOR) phosphorylation and VSMC marker expression. These changes are ascribed to increased palmitate levels and reduced oleate levels, changes that lead to reactive oxygen species (ROS) production and resulting AMP‐activated protein kinase (AMPK) activation. Notably, Elovl6 inhibition robustly induced the pluripotency gene Krüppel‐like factor 4 (KLF4) expression in VSMC, and KLF4 knockdown significantly attenuated AMPK‐induced phenotypic switching of VSMC, indicating that KLF4 is a bona fide target of AMPK.

**Conclusions:**

We demonstrate for the first time that dysregulation of Elovl6‐driven long‐chain fatty acid metabolism induces phenotypic switching of VSMC via ROS production and AMPK/KLF4 signaling that leads to growth arrest and downregulation of VSMC marker expression. The modulation of Elovl6‐mediated cellular processes may provide an intriguing approach for tackling atherosclerosis and postangioplasty restenosis.

## Introduction

Phenotypic modulation of vascular smooth muscle cells (VSMCs) is a critical process that regulates the progression of proliferative vascular disease including atherosclerosis and postangioplasty restenosis.^1^, ^2^, ^3^, ^4^ There have been extensive studies demonstrating that various growth factors, such as platelet‐derived growth factor (PDGF) and fibroblast growth factor‐2, promote phenotypic switching characterized by the downregulation of expression of differentiation marker genes such as smooth muscle α‐actin (SMα‐actin) and smooth muscle myosin heavy chain and the induction of proliferative capacity and proinflammatory gene expression.^2^, ^5^ There is now evidence that a significant fraction of VSMC within atherosclerotic plaques and cholesterol‐loaded cultured VSMC express markers of macrophages and mesenchymal stem cells as well as myofibroblasts.^6^, ^7^, ^8^ These findings argue that phenotypic switching of VSMC is more dynamically regulated than previously noted, and altered lipid metabolism within VSMC markedly contributes to phenotypic transitions of VSMC.

Elongation and desaturation are central steps in the de novo synthesis of long‐chain fatty acids (LCFAs) that determine their function and metabolic fate. The elongation of LCFA family member 6 (Elovl6) is by a rate‐limiting enzyme that mediates the elongation reaction of palmitate (C16:0) to stearate (C18:0), and stearoyl‐CoA desaturase (SCD) catalyzes the conversion of stearate to oleate (C18:1 n‐9).^9^ Mice deficient in Elovl6 (Elovl6^−/−^) are protected against diet‐induced insulin resistance despite their hepatosteatosis and obesity being similar to that in wild‐type (WT) mice.^10^ A more recent study reported that an atherogenic high‐fat diet induced hepatic inflammation, oxidative damage, and fibrosis in the liver, the hallmark features of nonalcoholic steatosis (NASH),^11^ but these were attenuated in Elovl6^−/−^ mice. Furthermore, Elovl6 deficiency in macrophages ameliorated foam cell formation.^12^ We previously demonstrated that Elovl6 inhibition in alveolar type II epithelial cells leads to severe pulmonary fibrosis.^13^ These findings suggest that Elovl6‐driven LCFA metabolism regulates a plethora of cellular function. However, VSMC phenotype in Elovl6^−/−^ mice remains unknown.

In the present study we investigated the effects of Elovl6 deficiency on VSMC phenotype in Elovl6^−/−^ mice and in cultured human aortic smooth muscle cells (HASMC) with small interfering RNA (siRNA)‐mediated Elovl6 knockdown. Our findings reveal that Elovl6 inhibition exerts profound effects on VSMC phenotype through an activation of AMP‐activated protein kinase (AMPK)/Krüppel‐like factor 4 (KLF4) signaling and strongly suggest a previously unrecognized mechanistic link between intracellular LCFA composition and VSMC phenotype.

## Methods

### Animal Model

C57BL/6 strain (WT) mice and Wistar rats were purchased from CLEA Japan Inc. Elovl6^−/−^ mice were a kind gift from Dr H. Shimano (Tsukuba University). These mice were intercrossed into Elovl6^−/−^ or Elovl6^−/+^ mice. Littermates were genotyped by PCR. The femoral artery of 10‐ to 12‐week‐old male WT mice (n=24, 24‐28 g) and Elovl6^−/−^ mice (n=23, 22‐26 g) was injured with a wire, as previously described,^14^ with some modifications.^15^ Briefly, a straight spring wire (0.38 mm in diameter, Cook Medical, Bloomington, IN) was inserted into the right femoral artery and pushed forward toward the iliac artery. The wire was left in place for 1 minute to denude and dilate the artery and then was removed. Blood flow was reconstituted after ligation of the profunda femoris branch. Mice were sacrificed 14 days after this injury, and the femoral arteries were isolated. Uninjured femoral arteries were isolated from sham controls. The carotid artery of rats was injured with a balloon as described previously.^16^ See Data S1 for further details. Animal experiments using these mice were approved by and performed according to the guidelines of the Committee of Experimental Animal Research of Gunma University (Permit Number: 10‐019).

### Cell Culture

VSMCs from the human aorta (HASMC) were purchased from Kurabo Ltd (New York, NY). See Data S1 for further details.

### RNA Isolation and Quantitative Real‐Time Reverse Transcription (qRT‐PCR)

Total RNA was extracted from the mouse aorta and cultured HASMC using ISOGEN regent (Takara Bio, Shiga, Japan) according to the manufacturer's protocol. See Data S1 for further details. All primer sequences are shown in Tables S1 and S2.

### Recombinant Adenovirus

Adenoviruses expressing LacZ (Ad‐LacZ) or Elovl6 (Ad‐Elovl6) were generated as described previously.^17^ HASMC were infected with Ad‐Elovl6 or Ad‐LacZ at a multiplicity of infection of 20 for 48 hours in preparation for the next experiments.

### Elongase Activity

Microsomal fatty acid elongation activity was assayed by measuring the incorporation of [2‐^14^C]malonyl‐CoA (Perkin Elmer, Shelton, CT) into exogenous acyl‐CoAs as described previously.^10^, ^13^

### [^3^H]Thymidine Uptake

The uptake of radiolabeled [^3^H]thymidine (Perkin Elmer, Shelton, CT) was assayed as described previously,^18^ with some modifications. See Data S1 for further details.

### Scratch Wound Healing Assay

The measurement of migration by the scratch mobility assay was performed as described previously.^19^ See Data S1 for further details.

### Boyden Chamber Assay

The Boyden chamber assay was performed as previously described.^19^ See Data S1 for further details.

### Fatty Acid Composition

Lipids from the mouse aorta and HASMC were extracted using the Bligh and Dyer method as described previously.^20^ See Data S1 for further details.

### Quantitation of ROS Measurement

For the quantitation of ROS levels, we used Hydrogen Peroxide/Peroxidase Assay Kit, Fluorometric (Cell Biolabs, San Diego, CA) according to the manufacturer's protocol. Red fluorescence (excitation 550 nm; emission 590 nm) was detected by microplate reader (Perkin Elmer, EnSpire, Shelton, CT).

### Statistical Analysis

All data are shown as the mean±standard error of the mean (SEM). A 2‐group comparison was performed using the Mann‐Whitney U‐test or unpaired Student t test, and a multiple‐group comparison was performed by a 1‐way ANOVA with Steel‐Dwass or Tukey‐Kramer multiple comparison tests. A *P*<0.05 was considered significant.

## Results

### Elovl6 Expression in Neointimal VSMC in Uninjured and Injured Femoral Artery in Wild‐Type Mice

**Figure 1:**
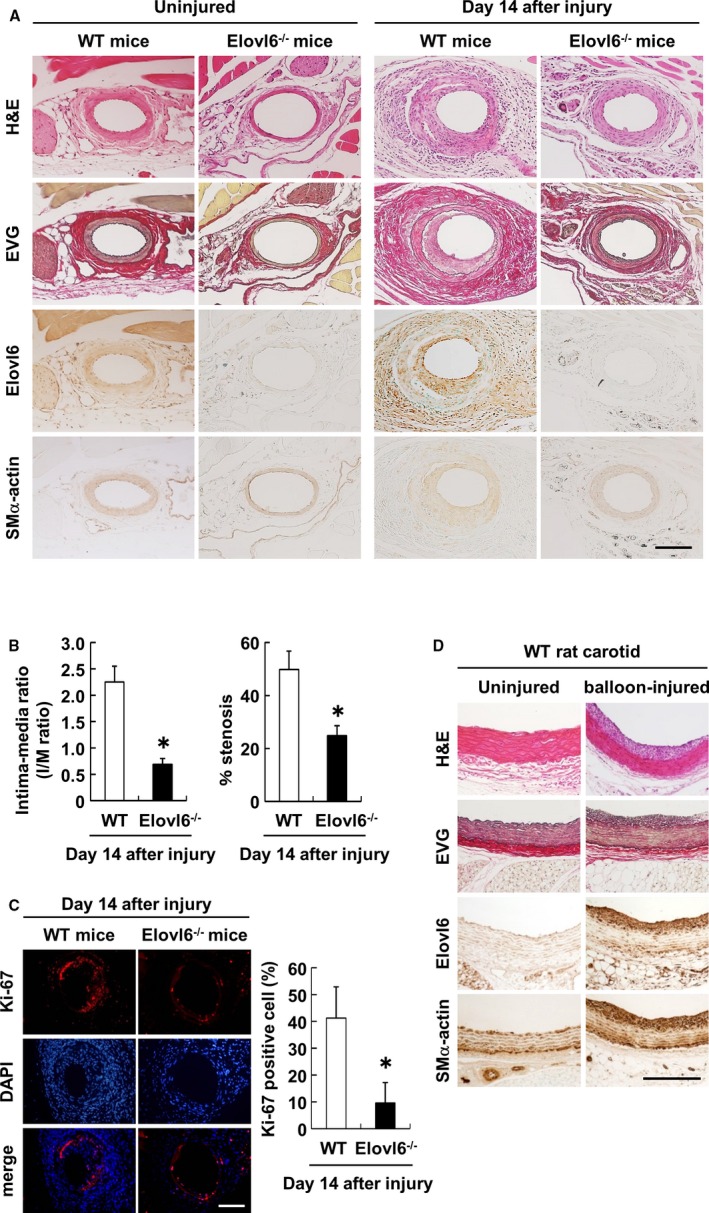
Neointimal hyperplasia after mechanical vascular injury is reduced in Elovl6^−/−^ mice. A, Representative section of H&E staining, EVG staining, and immunohistochemical staining with Elovl6 and SMα‐actin antibodies in mouse femoral artery 14 days after wire injury. The femoral arteries of 10‐ to 12‐week‐old male WT mice and Elovl6^−/−^ mice were injured with a wire. The expression of Elovl6 was colocalized with SMα‐actin‐positive cells in WT mice. The inhibition of neointimal hyperplasia was significantly greater in Elovl6^−/−^ mice than in WT mice. Scale bar=200 μm. B, The I/M ratio (left) and percentage stenosis area (right) were significantly lower in Elovl6^−/−^ mice (n=9) than in WT mice (n=10). C, Representative immunofluorescence staining with a Ki‐67 antibody (red) and DAPI (blue), and the number of Ki‐67‐positive cells per high‐power field in wire‐injured artery sections. The number of Ki‐67‐positive cells was markedly lower in Elovl6^−/−^ mice (n=9) than in WT mice (n=10). Scale bar=200 μm. D, Representative sections with H&E staining, EVG staining, and immunohistochemical staining with Elovl6 and SMα‐actin antibodies in the rat carotid artery subjected to balloon injury. Eight‐week‐old male Wistar rats were injured with a 2F balloon catheter. The expression of Elovl6 was colocalized with SMα‐actin‐positive cells in the uninjured WT rat carotid, and increased in the neointimal area of the balloon‐injured rat carotid. Scale bar=200 μm. All values are represented as the means. *P<0.05, measured by the Mann‐Whitney U‐test (B) or the Student t test (C). DAPI indicates 4′,6‐diamidino‐2‐phenylindole; Elovl6, elongation of long‐chain fatty acid family member 6; EVG, elastica van Gieson; H&E, hematoxylin‐eosin; I/M, intima/media; SMα‐actin, smooth muscle α‐actin; WT, wild‐type C57BL/6.

A mouse wire‐injury model, in which a straight spring wire was inserted into the femoral artery to denude and dilate the artery, was used in the present study. Immunohistochemistry revealed modest but discernible staining for Elovl6 in the medial layer of the uninjured femoral artery in WT mice. In contrast, the femoral artery 14 days after wire injury showed prominent positive staining for Elovl6 in the hypertrophic neointimal layer, in which SMα‐actin‐positive cells accumulated. Elovl6 staining was only modestly detected in the tunica media but was clearly detected in perivascular adipose tissue (Figure 1A).

### Mice Deficient in Elovl6 Display Reduced Neointima Formation After Vascular Injury

The femoral artery of Elovl6^−/−^ mice was subjected to wire injury to compare the neointima formation between WT and Elovl6^−/−^ mice. Hematoxylin‐eosin (H&E) and elastica van Gieson (EVG) staining showed that the thickness of the medial layer of uninjured femoral artery appeared thinner in Elovl6^−/−^ mice compared with that in WT mice. Immunohistochemistry revealed that neointima formation was markedly inhibited in Elovl6^−/−^ mice compared to WT mice, as assessed by H&E and EVG (Figure 1A). In addition, the semiquantitative intima/media (I/M) ratio and percentage stenosis assessment demonstrated that I/M ratio and percentage stenosis were significantly lower in Elovl6^−/−^ mice than in WT mice (Figure 1B). Furthermore, immunofluorescent Ki‐67 staining, a cell proliferation marker,^21^ showed that neointima of Elovl6^−/−^ mice had significantly fewer cells positive for Ki‐67 than a similar region in WT mice (Figure 1C). These results suggested that the knockout of Elovl6 inhibits neointima formation by repressing VSMC proliferation.

Immunohistochemistry of a rat carotid subjected to balloon injury confirmed the induction of Elovl6 expression in neointimal VSMC (Figure 1D). We also examined the expression of Elovl6 in the human coronary artery obtained by autopsy. Distinct immunoreactivity for Elovl6 was detected in the medial layer and diffuse intimal thickening area, in which SMα‐actin appears to be more strongly expressed (Figure S1).

### Elovl6 Knockdown Reduces VSMC Proliferation and Migration

**Figure 2:**
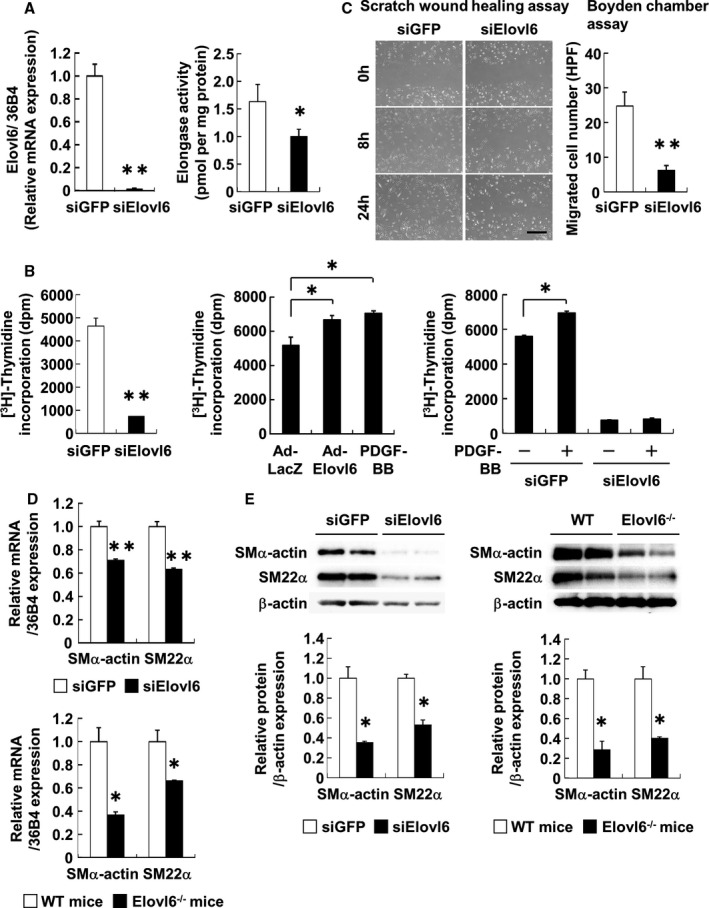
Inhibition of Elovl6 gene expression suppresses VSMC proliferation and migration. A, qRT‐PCR for Elovl6 mRNA expression and Elovl6 enzymatic activity in HASMC transfected with Elovl6 (n=6) or GFP siRNA (n=6). Elovl6 siRNA suppressed Elovl6 mRNA expression and activity more strongly than GFP siRNA. Specific activity was expressed as picomoles of radioactive [2‐^14^C]malonyl‐CoA incorporated into hydrophobic long‐chain fatty acid fractions by 1 mg of microsomal protein. B, The [^3^H]thymidine uptake assay in HASMC transfected with Elovl6 (n=6) or GFP siRNA (n=6), or adenovirus‐mediated overexpression of Elovl6 (n=6) or LacZ (n=6). The knockdown of Elovl6 expression markedly suppressed the incorporation of [^3^H]thymidine, whereas its overexpression induced the uptake of [^3^H]thymidine significantly more than the PDGF‐BB treatment (10 ng/mL, n=6) for 24 hours. The PDGF‐BB‐induced incorporation of [^3^H]thymidine was canceled by the siRNA‐mediated knockdown of Elovl6. C, Scratch assay and Boyden chamber assay in HASMC transfected with Elovl6 (n=6) or GFP siRNA (n=7). The knockdown of Elovl6 markedly suppressed cell migration. The number of migrated cells was counted under a light microscope. Scale bar=200 μm. D and E, qRT‐PCR for SMα‐actin and SM22α mRNA expression (D) and Western blot for protein expression (E) in HASMC transfected with Elovl6 (n=6) or GFP siRNA (n=6) and in the aorta of Elovl6^−/−^ mice (n=6) or WT mice (n=6). The mRNA levels of SMα‐actin and SM22α assessed by qRT‐PCR, normalized by mRNA levels of the nuclear single‐copy housekeeping gene 36B4, were significantly decreased by siRNA‐mediated knockdown of Elovl6 or in Elovl6^−/−^ mouse aorta. The levels of protein expression for SMα‐actin and SM22α were normalized by β‐actin. Each experiment was performed at least 3 times. All values are represented as the means±SEM of 3 experiments. *P<0.05, **P<0.01, as measured by the Mann‐Whitney U‐test, Student t test, or Tukey‐Kramer test. Elovl6 indicates elongation of long‐chain fatty acid family member 6; HASMC, human aortic smooth muscle cells; PDGF‐BB, platelet‐derived growth factor‐BB; SMα‐actin, smooth muscle α‐actin; SM22α, smooth muscle protein 22α; WT, wild‐type C57BL/6 mice.

To examine the role of Elovl6 in the regulation of VSMC proliferation and migration, Elovl6 was silenced by siRNA in cultured HASMC. We first verified that Elovl6 mRNA expression was efficiently downregulated (<1% of control si green fluorescent protein [GFP]‐transfected cell, *P*<0.01) as assessed by qRT‐PCR, and elongase activity was reduced to 66% compared with GFP siRNA (siGFP)‐transfected cells (*P*<0.05) (Figure 2A). We then examined the effects of Elovl6 knockdown on DNA synthesis by using [^3^H]thymidine incorporation assay. In Elovl6 siRNA (siElovl6)‐transfected HASMC, the incorporation of [^3^H]thymidine was markedly reduced compared with that in siGFP‐transfected control HASMC (Figure 2B). In order to confirm these results, we generated an adenovirus expressing Elovl6 (Ad‐Elovl6) (Figure S2). The results obtained showed that the overexpression of Elovl6 increased the incorporation of [^3^H]thymidine to a similar extent as PDGF‐BB treatment (Figure 2B). PDGF‐BB‐induced incorporation as well as baseline levels of [^3^H]thymidine incorporation were markedly diminished in siElovl6‐transfected HASMC (Figure 2B). These results suggested that Elovl6 had a marked impact on VSMC proliferation at baseline and played an indispensable role in mitogenic response elicited by PDGF‐BB. Together with the induction of Elovl6 gene expression by PDGF‐BB (Figure S3), these results suggested that Elovl6‐mediated fatty acid metabolism serves an intracellular cue for the proliferative response of VSMC to PDGF‐BB stimulation. Effects of Elovl6 on VSMC migration were examined using scratch wound healing assay. Inhibition of Elovl6 in HASMC resulted in a significant decrease in migration activity as determined by Boyden chamber assay (Figure 2C).

### Knockdown or Overexpression of Elovl6 Affects VSMC Marker Gene Expression

Because proliferation, migration, and differentiation are orchestrated through a shared cellular program, we determined whether Elovl6 was also involved in VSMC differentiation. We found that siRNA‐mediated knockdown of Elovl6 in cultured HASMC and the disruption of the Elovl6 gene in mice reduced mRNA and protein expression levels of the SMα‐actin and smooth muscle protein 22‐α (SM22α) genes, well defined VSMC markers (Figure 2D and 2E), while β‐actin protein levels were decreased. Such a decrease in β‐actin protein levels is probably due to the suppression of mTOR activity (see below) because the AMPK/mTOR pathway is known to regulate the expression of the genes relevant to cellular proliferation.^22^ In contrast, the adenovirus‐mediated overexpression of Elovl6 increased the expression of these genes (Figure S4A and S4B) in HASMC, suggesting that Elovl6‐driven fatty acid metabolism plays an important role in the regulation of VSMC differentiation.

### Elovl6 Regulates Cell Cycle Inhibitory Gene and Protein Expression

**Figure 3:**
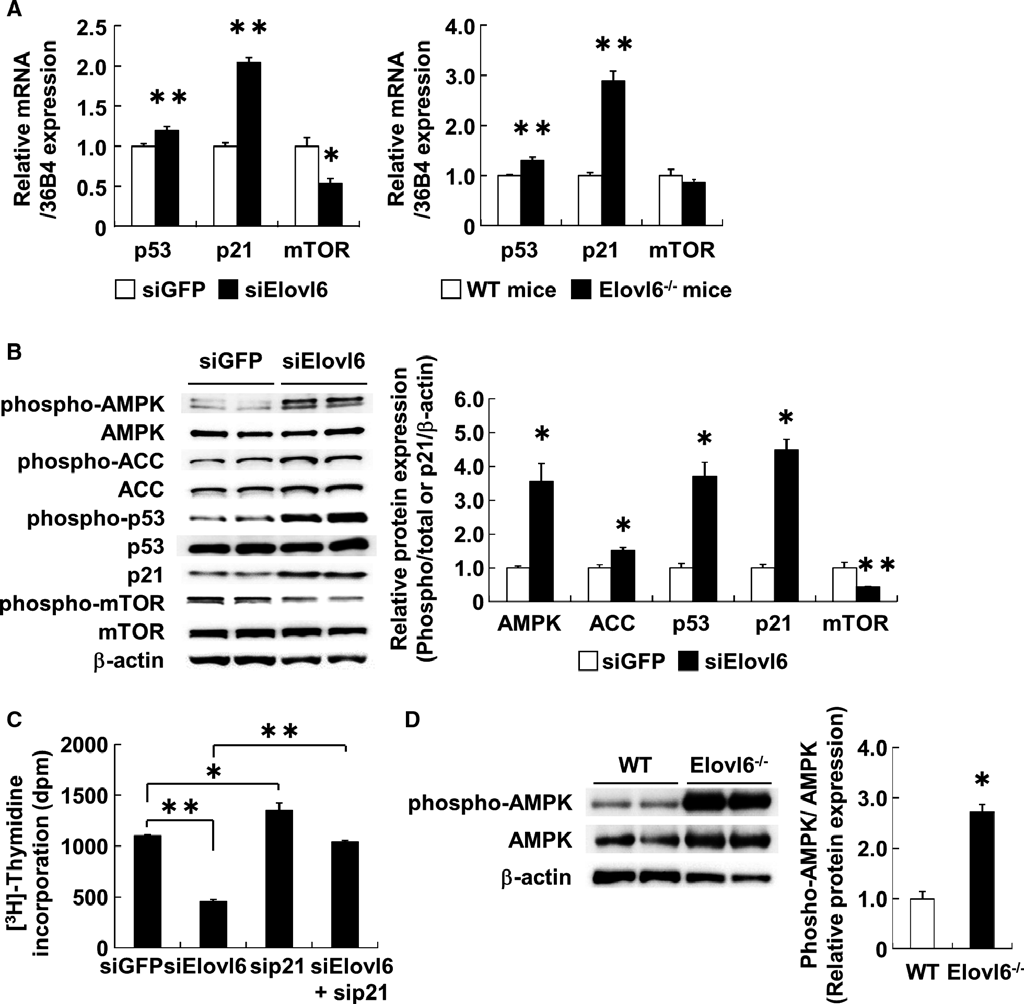
Depletion of Elovl6 expression modulates cell cycle regulators in HASMC. A, qRT‐PCR for p53, p21, and mTOR mRNA expression in HASMC transfected with Elovl6 (n=6) or GFP siRNA (n=8) and in the aorta of Elovl6^−/−^ mice (n=6) or WT mice (n=6). The expression levels of p53 and p21 mRNA, normalized by mRNA levels of 36B4, were significantly induced while that of mTOR mRNA was significantly decreased by siRNA‐mediated knockdown of Elovl6 or in the Elovl6^−/−^ mice aorta. B, Western blot analysis of each protein level involved in cell proliferation in HASMC. Protein levels were normalized by β‐actin. The knockdown of Elovl6 (n=5) significantly induced the phosphorylation of AMPK, ACC, and p53 and expression of p21 but reduced the phosphorylation of mTOR more than that in the siGFP‐transfected control cells (n=7). C, Effects of p21 gene silencing on the antiproliferative effects of the knockdown of Elovl6 in HASMC. Elovl6 siRNA‐mediated antiproliferative effects were canceled by p21 depletion (siGFP, siElovl6, sip21: n=6; siElovl6+sip21: n=5). D, Western blot analysis of the aorta of Elovl6^−/−^ mice (n=6) or WT mice (n=6). The phosphorylation of AMPK in the aorta was markedly increased in Elovl6^−/−^ mice compared to WT mice. Western blot data for phosphorylation of proteins were normalized by total proteins in the same samples and expressed as a fold increase from the mean level of siGFP or the WT mice. Each experiment was performed at least 3 times. All values are represented as the means±SEM of 3 experiments. *P<0.05, **P<0.01, as measured by the Mann‐Whitney U‐test (A, B, and D) or the Tukey‐Kramer test (C). ACC indicates acetyl‐CoA carboxylase; AMPK, AMP‐activated protein kinase; Elovl6, elongation of long‐chain fatty acid family member 6; HASMC, human aortic smooth muscle cells; mTOR, mammalian target of rapamycin; WT, wild‐type C57BL/6 mice.

To determine the mechanisms by which Elovl6 knockdown reduces VSMC proliferation, we examined the expression of mRNAs and proteins critical for cell proliferation in HASMC transfected with either siElovl6 or siGFP. The results obtained showed that knockdown of Elovl6 increased the p53 expression by 1.2‐fold and p21 expression by 2.0‐fold and decreased the mRNA levels of mammalian target of rapamycin (mTOR), a serine/threonine protein kinase that phosphorylates the binding protein essential for translational initiation and protein synthesis, by 45% (Figure 3A). Consistent with these results, the mRNA levels of p53 and p21 in the aorta were 1.3‐ and 2.9‐fold higher, respectively, while those of mTOR were comparable between Elovl6^−/−^ and WT mouse aorta (Figure 3A). Knockdown of Elovl6 induced the phosphorylation of p53 tumor suppressor and protein expression of cyclin‐dependent kinase inhibitor p21 (Figure 3B). An increase in the expression of p21 is likely due to the activation of p53 given that the p21 gene is a direct target of p53.^23^ The phosphorylation of mTOR was clearly decreased in siElovl6‐transfected HASMC (Figure 3B).

Conversely, the overexpression of Elovl6 increased mTOR mRNA levels (Figure S4C). To further analyze the role of p21 in HASMC with silenced Elovl6, siRNA for p21 was transfected with or without siRNA for Elovl6, and the effects of the silencing of p21 on thymidine uptake were evaluated. The p21 siRNA treatment resulted in an increase in the uptake of thymidine, and, more importantly, the repressed thymidine uptake in cells with Elovl6 knockdown was completely reversed by p21 siRNA treatment (Figure 3C). These results indicate that the Elovl6 knockdown reduces cell proliferation largely through the induction of p21 expression.

### Elovl6 Deficiency Induces AMPK Activity in VSMC

AMP‐activated protein kinase (AMPK), a heterotrimeric serine/threonine protein kinase that is primarily activated in response to nutrients and energetic stress,^24^, ^25^, ^26^ has been shown to regulate cell proliferation via a p53 and p21 pathway in response to vascular injury.^27^, ^28^ To explore the effects of Elovl6 deficiency on AMPK activation, we performed Western blot analysis by using the antibody against phosphopeptides containing Thr‐172 of the α subunit of human AMPK. Results showed that knockdown of Elovl6 markedly induced the phosphorylation of AMPK in HASMC and that the total protein levels of AMPK were comparable between siGFP‐ and siElovl6‐transfected cells. The p21 protein levels were increased while β‐actin protein levels were decreased (Figure 3B). Consistently, Elovl6^−/−^ mice exhibited significantly increased phosphorylated AMPK levels and slightly decreased β‐actin levels compared to WT mice (Figure 3D). In contrast, the overexpression of Elovl6 decreased phosphorylation of AMPK (Figure S4D).

AMPK activation has been shown to mediate an increase in fatty acid oxidation via acetyl‐CoA carboxylase (ACC) phosphorylation at Ser‐78, which leads to inactivation of ACC activity and reduction of malonyl‐CoA synthesis, in the setting of metabolic stress.^29^ We found that ACC phosphorylation is associated with AMPK activation in HASMC with Elovl6 knockdown (Figure 3B), providing evidence that AMPK activity is indeed increased in these cells. Because inhibition of ACC and subsequent decrease in malonyl‐CoA production lead to a suppression of lipogenesis and favor fatty acid oxidation through an induction of carnityl palmitoyltransferase (CPT) shuttle system,^30^ it is likely that Elovl6‐deficient VSMC has reduced synthesis and increased oxidation of LCFA. As expected, FA oxidation rate, as assessed by using [^14^C]palmitic acid, was higher in VSMC transfected with siElovl6 in comparison with siGFP‐transfected VSMC. Furthermore, the mRNA levels of peroxisome proliferator‐activated receptor (PPAR)α, the nuclear receptor playing a crucial role as an activator of genes involved in fatty acid oxidation,^31^ were increased in HASMC with Elovl6 knockdown as compared with siGFP‐transfected cells (data not shown).

### Elovl6 Knockdown Alters LCFA Composition in VSMC In Vivo and In Vitro

**Figure 4:**
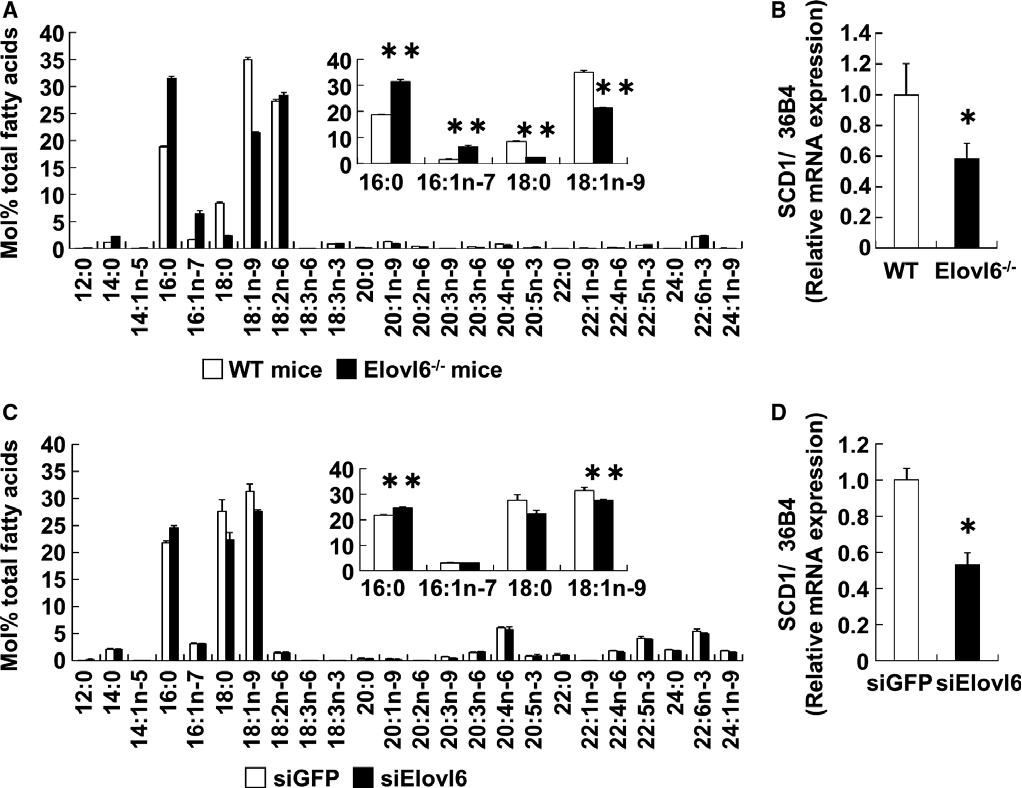
Elovl6 depletion modulates fatty acid composition in vivo and in vitro. A and B, Fatty acid composition of a lipid extract (A) and qRT‐PCR for SCD1 gene expression (B) from the aorta of Elovl6^−/−^ (n=5) or WT mice (n=4). The relative amounts of palmitate (C16:0) were higher, whereas those of oleate (C18:1 n‐9) were lower in Elovl6^−/−^ mice than in WT mice (A). SCD1 mRNA levels, normalized by mRNA levels of 36B4, were lower in Elovl6^−/−^ mice (n=5) than in WT mice (n=4) (B). C and D, Fatty acid composition of a neutral lipid extract (C) and qRT‐PCR for SCD1 gene expression (D) in HASMC transfected with Elovl6 (n=5) or GFP siRNA (n=6). Along with in vivo data, the relative amounts of palmitate were significantly higher, whereas those of oleate (C) and SCD1 mRNA levels, normalized by mRNA levels of 36B4 (D), were lower with the knockdown of Elovl6 than with siGFP transfection. mRNAs of the WT mice (B) and siGFP (D) control group were normalized to a value of 1, and mRNA levels in Elovl6^−/−^ mice or Elovl6 siRNA cells are shown relative to the control level. Each experiment was performed at least 3 times. All values are represented as the means±SEM of 3 experiments. *P<0.05, **P<0.01, as measured by the Mann‐Whitney U‐test. Elovl6 indicates elongation of long‐chain fatty acid family member 6; HASMC, human aortic smooth muscle cells; SCD1, stearoyl‐CoA desaturase 1; WT, wild‐type C57BL/6 mice.

To determine the effect of Elovl6 knockdown on LCFA metabolism, we examined the composition of LCFA in lipids extracted from the aorta of Elovl6^−/−^ and WT mice. Saturated palmitate (C16:0) levels were higher, and monounsaturated oleate (C18:1 n‐9) levels were lower in Elovl6^−/−^ mice than in WT mice (Figure 4A). The qRT‐PCR revealed that the aortic expression of the gene for SCD1, a rate‐limiting enzyme that converts saturated fatty acids (SFAs) to monounsaturated fatty acids, was lower in Elovl6^−/−^ mice than in WT mice (Figure 4B) (60% of the control value, *P*<0.05).

Alterations of the composition of LCFA and reductions of the expression of SCD1 in the aorta of Elovl6^−/−^ mice were recapitulated by Elovl6 siRNA treatment in vitro (Figure 4C and 4D). These results also indicate that metabolic phenotype such as a lower oleate (C18:1 n‐9) levels with an Elovl6 knockdown may be partially due to a decrease in SCD1 activity. In contrast, the overexpression of Elovl6 in HASMC decreased palmitate and palmitoleate (C16:1 n‐7) levels and increased oleate levels (Figure S5A), whereas the mRNA levels of SCD1 were similar in HASMC transduced with Ad‐Elovl6 or Ad‐LacZ (Figure S5B). Taken together, these results indicate that Elovl6 in VSMC plays a major role in determining the fatty acid composition of LCFA in VSMC.

### Effects of Palmitate and Oleate on Cell Proliferation and Cell Cycle Regulators

**Figure 5:**
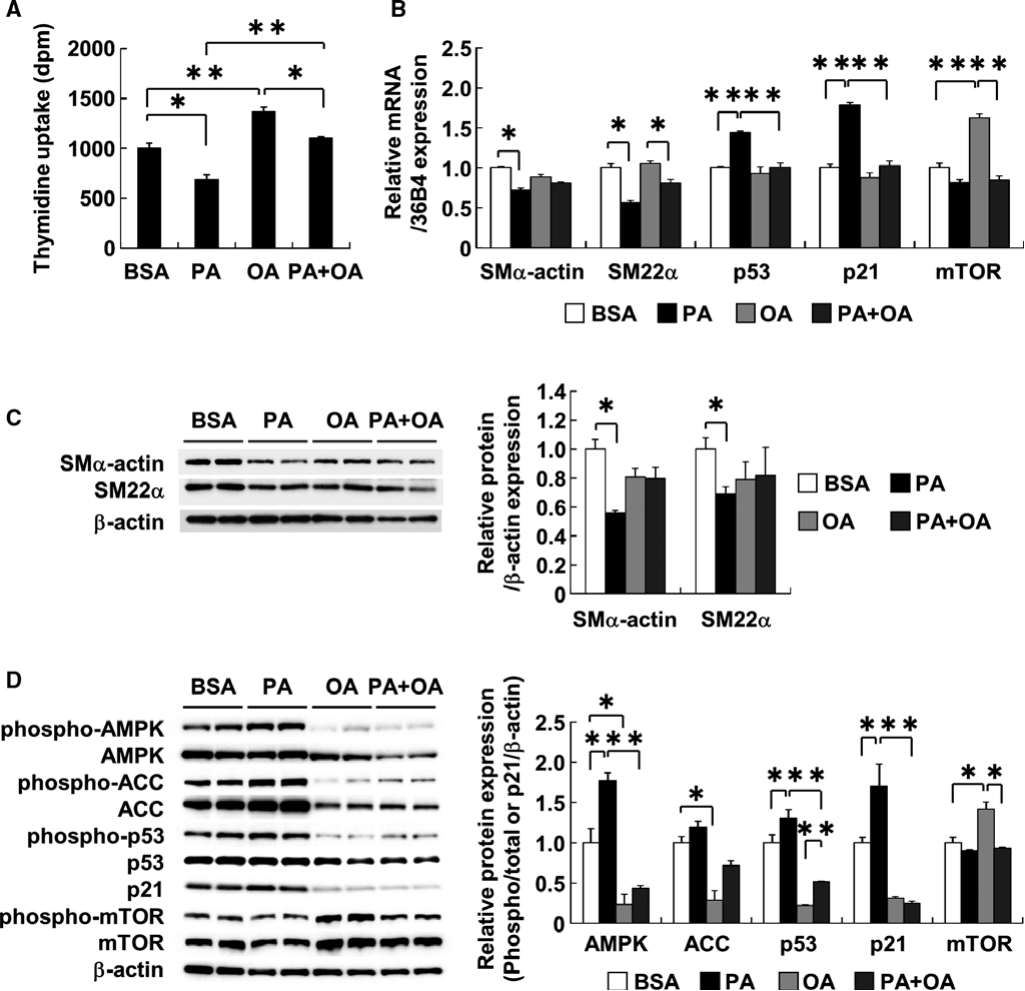
Palmitate suppresses cell proliferation by altering cell cycle regulatory proteins in HASMC. A, [^3^H]Thymidine uptake assay in HASMC treated with PA or OA. PA (250 μmol/L) treatment significantly suppressed, whereas OA (250 μmol/L) treatment induced, the incorporation of [^3^H]thymidine (BSA, OA: n=6; PA: n=8). Simultaneous treatment with OA significantly inhibited PA‐induced antiproliferative effects (PA+OA: n=6). B, qRT‐PCR for SMα‐actin, SM22α, p53, p21, and mTOR mRNA levels, normalized by mRNA levels of 36B4, in HASMC treated with PA or OA (BSA: n=7; PA, OA, PA+OA: n=6). The mRNA levels for SMα‐actin and SM22α were significantly decreased, whereas that of p21 and p53 mRNA levels were increased by PA treatment. In contrast, mTOR mRNA levels were significantly increased by OA treatment. mRNA levels in the siGFP control group were normalized to a value of 1, and those in cells treated with various stimuli are shown relative to the control level. C and D, Western blot analysis of each protein level involved in cell proliferation in HASMC treated with PA or OA (BSA: n=8; PA, OA, PA+OA: n=6). PA markedly induced the phosphorylation of AMPK, ACC, and p53 and expression of p21, whereas OA induced the phosphorylation of mTOR. Simultaneous treatment with PA and OA mutually suppressed the induction of protein expression. Furthermore, treatment of PA decreased SMα‐actin and SM22α protein expression. Western blot data for phosphorylation of proteins were normalized by total protein amount in the same samples and expressed as a fold increase from the mean level of the BSA group; p21 protein levels were normalized by β‐actin protein levels. Each experiment was performed at least 3 times. All values are represented as the means±SEM of 3 experiments. *P<0.05, **P<0.01, as measured by the Tukey‐Kramer test (A and B) or the Steel‐Dwass test (C and D). ACC indicates acetyl‐CoA carboxylase; AMPK, AMP‐activated protein kinase; BSA, fatty acid‐free bovine serum albumin; HASMC, human aortic smooth muscle cells; mTOR, mammalian target of rapamycin; OA, oleic acid; PA, palmitic acid; SM22α, smooth muscle protein 22α; SMα‐actin, smooth muscle α‐actin.

Given the data presented above, we examined the effects of palmitate and oleate on VSMC proliferation and cell cycle regulatory gene expression. Palmitate treatment reduced the incorporation of [^3^H]thymidine by 30% (*P*<0.05), whereas oleate treatment increased it by 20% (*P*<0.01) (Figure 5A). The qRT‐PCR analysis also revealed that palmitate suppressed the SMα‐actin (72%, *P*<0.05), SM22α (56%, *P*<0.05), p53 (1.4‐fold, *P*<0.01), and p21 (1.8‐fold, *P*<0.01) mRNA levels, whereas oleate increased the mTOR mRNA levels (1.6‐fold, *P*<0.01) (Figure 5B). Western blot analysis also showed that palmitate treatment decreased protein levels of SMα‐actin and SM22α (Figure 5C). Furthermore, palmitate treatment significantly induced the phosphorylation of AMPK, ACC, and p53, increased the expression of p21, and decreased the phosphorylation of mTOR (Figure 5D). In contrast, oleate treatment decreased total protein levels of AMPK, ACC, and p53 and p21, and increased total mTOR levels, as previously reported,^32^, ^33^, ^34^, ^35^ and as such, levels of phosphorylated form of AMPK, ACC, and p53 were decreased, and levels of phosphorylated form of mTOR were increased (Figure 5D). A simultaneous stimulation with palmitate and oleate consistently showed that each of these 2 LCFAs antagonizes the effects of the other (Figure 5A through 5D).

### Elovl6 Knockdown Induces KLF4 Expression in VSMC

**Figure 6:**
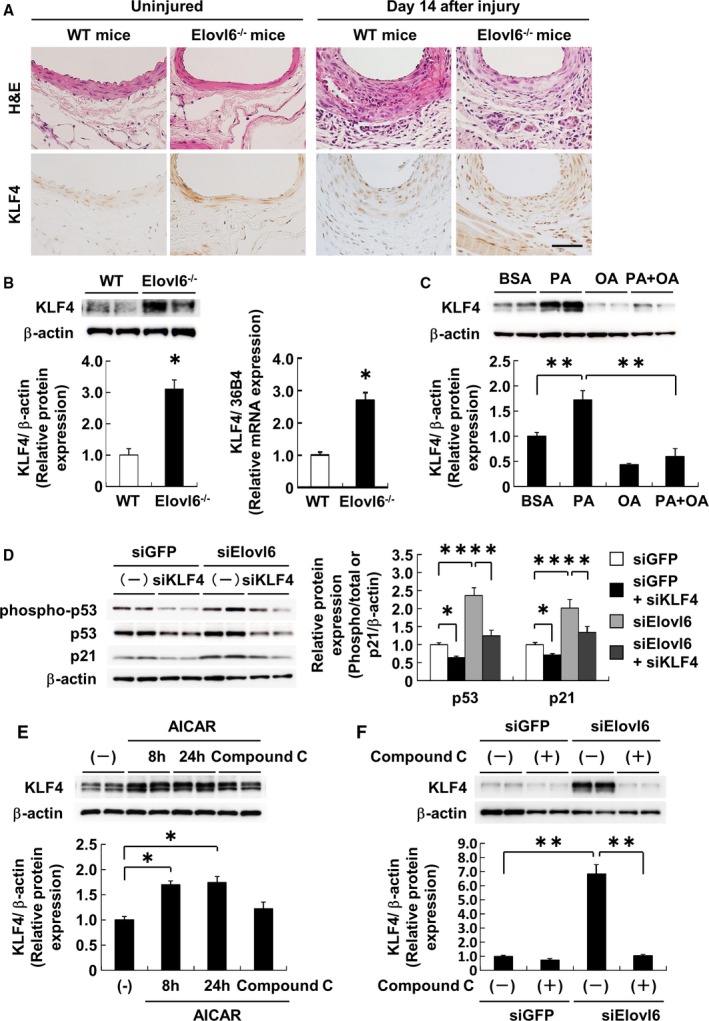
Elovl6 deficiency directly induces KLF4 expression through AMPK activation. A, Immunohistochemical staining with an anti‐KLF4 antibody in femoral artery sections from Elovl6^−/−^ or WT mice. Expression of KLF4 was significantly increased in the medial layer of the aorta of Elovl6^−/−^ mice compared to WT mice before or after wire injury. Scale bar=200 μm. B, Western blot and qRT‐PCR analyses of KLF4 expression in the aorta of Elovl6^−/−^ mice (n=6). KLF4 expression was significantly induced in the Elovl6^−/−^ mice aorta compared to WT mice (n=6). C, Western blot analysis of KLF4 in HASMC treated with PA or OA. KLF4 was significantly increased by PA treatment but decreased by OA treatment (BSA, PA: n=8; OA, PA+OA: n=6). D, Effects of KLF4 gene silencing on Elovl6 siRNA‐induced cell cycle regulatory proteins in HASMC by Western blot analysis. Elovl6 siRNA‐induced phospho‐p53 or p21 protein levels were suppressed by the depletion of KLF4 (siGFP, siElovl6, siGFP, or siElovl6+siKLF4: n=6). E, Western blot analysis of AICAR (1 mmol/L, n=6) and Compound C (10 μmol/L, n=6) stimulations in HASMC. Treatment of AICAR induced the KLF4 expression in a time‐dependent manner, but costimulation with Compound C (n=6) reduced the AICAR‐induced expression of KLF4. F, Western blot analysis of KLF4 in GFP siRNA (n=5)‐ or Elovl6‐siRNA (n=6)‐transfected HASMC with or without Compound C. Compound C abrogated the Elovl6 siRNA‐induced expression of KLF4. The mRNA levels determined by qRT‐PCR were normalized by 36B4, and protein levels determined by Western blot analysis were normalized by β‐actin levels. Each experiment was performed at least 3 times. All values are represented as the means±SEM of 3 experiments. *P<0.05, **P<0.01, as measured by the Mann‐Whitney U‐test (B), or the Steel‐Dwass test (C through F). AICAR indicates 5‐aminoimidazole‐4‐carboxamide ribonucleotide; BSA, fatty acid‐free bovine serum albumin; Elovl6, elongation of long‐chain fatty acid family member 6; H&E, hematoxylin‐eosin; HASMC, human aortic smooth muscle cells; KLF4, Krüppel‐like factor 4; OA, oleic acid; PA, palmitic acid; WT, wild‐type C57BL/6 mice.

Krüppel‐like factor 4 (KLF4), a reprogramming factor that promotes induced pluripotent stem (iPS) cell formation,^36^ controls the expression of VSMC marker genes.^37^, ^38^ KLF4 induces the expression of p21 in concert with the activation of p53 and as a result decreases the proliferation of VSMC.^38^ We performed a series of experiments to test whether KLF4 is involved in the suppression of proliferation of Elovl6 siRNA‐transfected HASMC. Immunohistochemistry revealed that KLF4 expression was more evident in Elovl6^−/−^ mice than in WT mice in both uninjured and wire‐injured aorta (Figure 6A). Western blot and qRT‐PCR analyses using protein and RNA extracts from aorta, respectively, confirmed these findings (Figure 6B). In addition, Elovl6 knockdown led to a robust elevation in KLF4 expression at both protein and mRNA levels (Figure S6A and S6B). Overexpression of Elovl6 suppressed the KLF4 protein and mRNA levels (Figure S6C and S6D), substantiating the above findings. Of note, exogenous palmitate significantly upregulated KLF4 protein and mRNA levels, and oleate blunted these effects (Figure 6C and Figure S6E), further supporting the findings that KLF4 expression is regulated by Elovl6‐driven fatty acid metabolism.

### KLF4 Knockdown Mitigates Upregulation of p53 and p21 in Elovl6‐Deficient VSMC

We next examined the cell cycle regulatory gene expression and cell proliferation in HASMC with Elovl6 knockdown. After confirming substantial depletion of KLF4 (Figure S7A and S7B), we performed qRT‐PCR analysis. Simultaneous knockdown of KLF4 partly rescued the suppressed proliferation in siElovl6‐transfected HASMC (Figure S7C). Consistent with these results, knockdown of KLF4 abrogated the increase in phospho‐p53 and p21 protein levels as well as p53 and p21 mRNA levels observed in HASMC with Elovl6 knockdown (Figure 6D and Figure S7D). These results suggest that KLF4 mediates in part an induction of p21 and p53 expression observed in HASMC with Elovl6 deficiency.

### KLF4 Knockdown Affects SMC Marker Gene Expression in a Context‐Dependent Manner

KLF4 has been shown to act as potent repressor of VSMC gene transcription in cultured VSMCs through multiple mechanisms including the repression of function and expression of muscle‐restricted transcription coactivator myocardin.^38^, ^39^, ^40^ Thus, it is anticipated that KLF knockdown enhances VSMC marker gene expression. Unexpectedly, however, we found that mRNA expression of SMα‐actin and SM22α was significantly reduced in HASMC with KLF4 knockdown (Figure S8A). Nevertheless, Western blot analysis revealed that the protein levels of SMα‐actin and SM22α were not changed in HASMC transfected with siKLF4 (Figure S8B). The most likely explanation for these apparently contradictory results would be that experimental conditions may affect the role of KLF4 on SMC gene expression, given that KLF4 only transiently binds to the SMC promoters at an early time point after KLF4 induction.^38^ Indeed, we found that siRNA‐mediated Elovl6 or KLF4 knockdown increased SMα‐actin or SM22α expressions at an early time point (8‐24 hours after transfection of siRNA); however, Elovl6 or KLF4 knockdown markedly suppressed SMC marker gene expression 48 hours after transfection (Figure S8C).

### AMPK Directly Activates KLF4 Expression in VSMC In Vitro

To determine whether AMPK activates KLF4 expression, we examined the effects of a pharmacological AMPK activator (5‐aminoimidazole‐4‐carboxamide ribonucleotide; AICAR) or inhibitor (Compound C) on KLF4 protein expression. AICAR significantly induced KLF4 protein levels in a time‐dependent manner, and Compound C diminished this effect (Figure 6E). Of importance, Compound C completely abrogated an increase in KLF4 protein levels observed in HASMC with Elovl6 knockdown (Figure 6F). In contrast, KLF4 knockdown had no effects on phosphorylation of AMPK (Figure S9). Taken together, these results identify KLF4 as a bona fide direct target of AMPK.

### AMPK Phosphorylation Is Induced by Oxidative Stress

**Figure 7:**
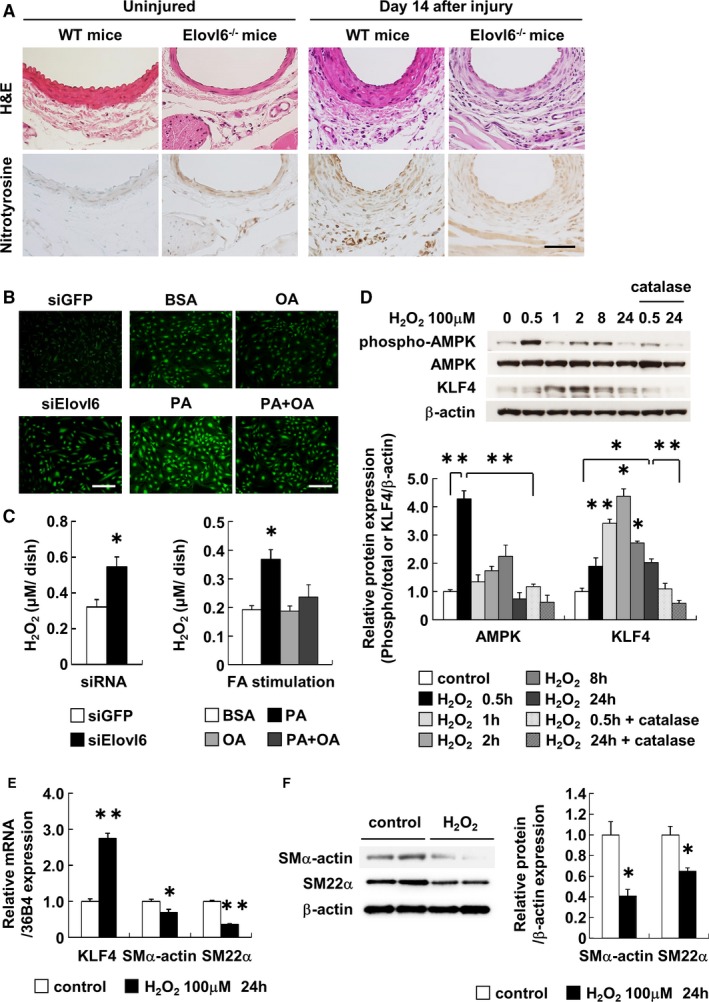
ROS generation induces phosphorylation of AMPK and KLF4 expression in VSMC. A, Immunohistochemical staining with an antibody against nitrotyrosine in femoral artery sections from Elovl6^−/−^ or WT mice. The nitrotyrosine‐positive cells were significantly increased in the medial layer of the aorta of Elovl6^−/−^ mice compared to WT mice before or after wire injury. Scale bar=200 μm. B, Intracellular ROS production was detected using oxidant‐sensitive fluorogenic probe CM‐H_2_ DCFDA in HASMC. The siRNA‐mediated Elovl6 knockdown or stimulation with PA (250 μmol/L) increased ROS levels in comparison with siGFP or BSA. Costimulation of OA (250 μmol/L) significantly reduced PA‐induced ROS generation. Scale bar=200 μm. C, fluorometric assay showed increased H_2_O_2_ generation in HASMC transfected with Elovl6 siRNA (siGFP, siElovl6: n=6) or treated with PA (BSA, PA, OA, PA+OA: n=6). D, Western blot analysis of AMPK and KLF4 in HASMC treated with 100 μmol/L H_2_O_2_. Phosphorylation of AMPK was normalized by total AMPK protein amount, and KLF4 protein levels were normalized by β‐actin levels. Relative protein expression was expressed as a fold increase from the mean level of the controls. Exposure to H_2_O_2_ (n=3 per group) significantly induced phosphorylation of AMPK and KLF4 expression. Costimulation with catalase (1 unit, n=3 per group) significantly reduced H_2_O_2_ induction of these expressions. E, qRT‐PCR for KLF4, SMα‐actin, and SM22α mRNA levels in HASMC treated with 100 μmol/L H_2_O_2_ for 24 hours (control: n=6; H_2_O_2_: n=8). KLF4 mRNA levels, normalized by mRNA levels of 36B4, were significantly increased, whereas SMα‐actin and SM22α mRNA levels were decreased by H_2_O_2_ treatment. F, Western blot analysis also showed that exposure of HASMC to H_2_O_2_ decreased protein expression of SMα‐actin and SM22α, normalized by β‐actin levels (control, H_2_O_2_: n=6). Each experiment was performed at least 3 times. All values are represented as the means±SEM of 3 experiments. *P<0.05, **P<0.01, as measured by the Tukey‐Kramer test (C), Steel‐Dwass test (D), or Mann‐Whitney U‐test (E and F). AMPK indicates AMP‐activated protein kinase; BSA, fatty acid‐free bovine serum albumin; CM‐H_2_ DCFDA, 5‐(6)‐chloromethyl‐2′,7′‐dichlorodihydrofluorescein diacetate acetyl ester; Elovl6, elongation of long‐chain fatty acid family member 6; H&E, hematoxylin‐eosin; H_2_O_2_, hydrogen peroxide; HASMC, human aortic smooth muscle cells; KLF4, Krüppel‐like factor 4; OA, oleic acid; PA, palmitic acid; ROS, reactive oxygen species; SM22α, smooth muscle protein 22α; SMα‐actin, smooth muscle α‐actin; WT, wild‐type C57BL/6 mice.

To explore the mechanisms by which Elovl6 deficiency leads to an induction of AMPK phosphorylation in VSMC, we examined the reactive oxygen species (ROS) production in Elovl6^−/−^ mice. Immunohistochemistry revealed that nitrotyrosine immunoreactivity, used as a marker of oxidative stress, was fairly detectable in Elovl6^−/−^ mice but poorly detectable in WT mice (Figure 7A). At 14 days after wire injury, however, nitrotyrosine immunoreactvity was clearly enhanced in both WT and Elovl6^−/−^ mice (Figure 7A). Furthermore, we found that siRNA knockdown of Elovl6 induced ROS production as assessed by using CM‐H_2_DCFDA probe in HASMC (Figure 7B). In addition, palmitate induced ROS production, and oleate inhibited this palmitate‐induced ROS production in HASMC (Figure 7B). Quantitation using a fluorometric assay, which primarily detects production of H_2_O_2,_ showed that Elovl6 knockdown and exposure to palmitate increased production of H_2_O_2_ in HASMC by 1.7‐ and 1.9‐fold, respectively, compared with controls (Figure 7C). To explore whether mitochondria could be involved in ROS production, we measured mitochondria‐derived superoxide levels by using mitoSOX fluorescent probes (Life Technologies, Carlsbad, CA). However, we could hardly detect the difference in signal intensity among siElovl6‐transfected HASMC, palmitate‐treated HASMC, and control cells, suggesting that excessive superoxide is not likely to be generated in mitochondria in either siElovl6‐transfected or palmitate‐treated HASMC.

We found that exposure of HASMC to hydrogen peroxide (H_2_O_2_ 100 μmol/L) resulted in induced AMPK phosphorylation and subsequently increased KLF4 expression (Figure 7D). Catalase, an antioxidant that eliminates H_2_O_2_, prevented H_2_O_2_‐induced AMPK phosphorylation and KLF4 expression in HASMC (Figure 7D). Furthermore, exposure of HASMC to H_2_O_2_ increased KLF4 mRNA and decreased SMα‐actin and SM22α mRNA levels (Figure 7E). Western blot analysis also showed that exposure of HASMC to H_2_O_2_ decreased protein levels of SMα‐actin and SM22α (Figure 7F). These results suggest that ROS is an important mediator for the induction of AMPK phosphorylation and the resulting KLF4 expression in Elovl6‐deficient VSMC.

## Discussion

**Figure 8:**
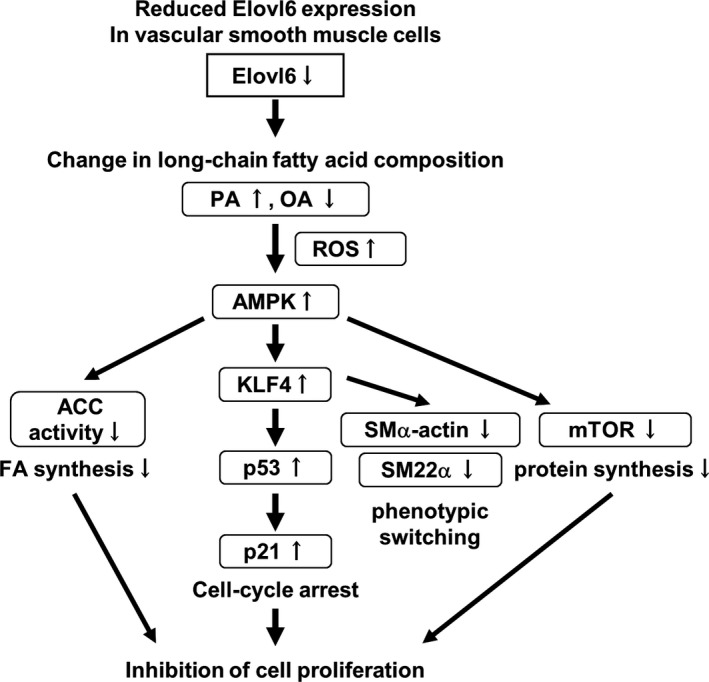
Schematic diagram of the molecular mechanisms for phenotypic modulation of VSMC in Elovl6‐deficient VSMC. A reduction in the expression of Elovl6 in VSMC induces change in the fatty acid composition; C16 PA is increased, and C18 OA is decreased, in vivo and in vitro. These changes in FA composition induce ROS production that leads to AMPK activation, an increase in KLF4, p53, and p21 expression, decreases in SMα‐actin, SM22α, and mTOR expression, and a decrease in ACC activity. Thus, Elovl6‐mediated derangement of FA composition in VSMC induces phenotypic switching of VSMC. ACC indicates acetyl‐CoA carboxylase; AMPK, AMP‐activated protein kinase; Elovl6, elongation of long‐chain fatty acid family member 6; FA, fatty acid; KLF4, Krüppel‐like factor 4; mTOR, mammalian target of rapamycin; OA, oleic acid; PA, palmitic acid; ROS, reactive oxygen species; SM22α, smooth muscle protein 22α; SMα‐actin, smooth muscle α‐actin; VSMC, vascular smooth muscle cells.

In the present study we examined the role of Elovl6‐mediated LCFA metabolism in the regulation of VSMC phenotypic switching in vivo and in vitro. To summarize, results shown here are depicted in Figure 8. Elovl6 deficiency causes an increase in intracellular palmitate and a decrease in oleate, both of which contribute to ROS production and promote phosphorylation and activation of AMPK. As a consequence of AMPK activation, KLF4 is induced to increase p53 and p21 expression, ACC is phosphorylated, and mTOR is dephosphorylated. Our findings strongly indicate the pivotal role of Elovl6‐driven fatty acid metabolism in the regulation of proliferation and differentiation of VSMC, 2 pathophysiological processes that determine VSMC phenotype.

One of the seminal findings in this study is that KLF4 expression is robustly induced in Elovl6^−/−^ mouse aorta and VSMC with siRNA knockdown of Elovl6. Elovl6 siRNA‐transfected VSMC is characterized by reduced proliferation and migration as well as reduced expression of VSMC markers. More importantly, these changes are likely to be at least partly ascribed to KLF4 induction because KLF4 knockdown mitigates activation of the cell cycle inhibitory genes p53 and p21 in Elovl6‐deficient VSMC. These results are consistent with the previous reports demonstrating that KLF4 induces cell cycle arrest.^38^, ^41^, ^42^, ^43^, ^44^

Of note, induction of KLF4 expression by Elovl6 knockdown is dependent on AMPK activation, based on the results that Compound C, a specific inhibitor of AMPK, completely abrogated KLF4 induction. Our data also indicate that KLF4 is required for AMPK‐induced p21 expression. Collectively, our results demonstrate that KLF4 is one of the primary genes that regulate cellular response to AMPK activation.

It is notable that VSMC transfected with siElovl6 displays downregulation of SMC marker gene expression along with decreased proliferation and migration activities, given that the loss of SMC marker gene expression is generally coupled with the increased proliferation and migration during phenotypic transition.^2^ Our findings that the AMPK/KLF4 axis plays a central role in phenotypic transition in VSMC with Elovl6 knockdown prompt us to speculate that the phenotype of Elovl6‐deficient VSMC may be relevant to cellular senescence‐associated phenotype. Cellular senescence is observed in most mammalian cells in the setting of a variety of stresses including alterations in intracellular metabolism.^45^ Emerging evidence indicates that AMPK activation and resulting p53‐p21 axis activation promote senescent phenotype, the hallmarks of which are the irreversible loss of proliferative potential and acquisition of altered differentiated function.^46^ We found that Elovl6 knockdown induces apoptosis as assessed by the activation of caspase 3/7 to some extent (data not shown), suggesting that altered Elovl6‐mediated LCFA metabolism induces both apoptosis and perhaps a state of senescence. Further work will be warranted to define such previously unrecognized phenotypes.

A recent SMC lineage‐tracing study demonstrates that substantial numbers of macrophage‐like cells within the atherosclerotic plaques are derived from VSMC in apolipoprotein E (apoE)‐knockout mice and in humans.^8^ In addition, VSMC‐specific conditional knockout of KLF4 results in decreased lesion size, increased plaque stability, and a marked reduction in the number of VSMC‐derived macrophage‐like cells. Of particular interest in that study, in addition to an increase in macrophage and proinflammatory marker expression, KLF4 is induced in response to cholesterol loading in cultured VSMC. These observations led us to speculate that KLF4 is activated by metabolic stress triggered by perturbed intracellular lipid metabolism, such as cholesterol loading and altered LCFA composition, and if so, the AMPK/KLF4 axis plays a principal role in passing the metabolic stress on to the machinery regulating the phenotype of VSMC. Furthermore, the finding that an intracellular dysmetabolic state regulates KLF4 expression might best be viewed as evidence indicating that nutrient metabolism, cellular behavior, and cell cycle are intimately interconnected.^28^

A key question is how Elovl6 deficiency within VSMC results in a marked activation of AMPK. The classical view is that AMPK, a highly conserved heterotrimer comprising an α‐catalytic subunit with βγ‐regulatory subunits, responds to a reduction in the energy charge, switches off anabolic pathways such as fatty acid, triglyceride, and cholesterol synthesis, and switches on catabolic pathways that generate ATP, such as fatty acid oxidation and glycolysis.^24^, ^25^, ^26^ However, activation of AMPK in Elovl6‐deficient VSMC is not likely due to reduced cellular ATP content because exposure of cultured HASMC with palmitate recapitulated the response to Elovl6 knockdown. Thus, we propose that specific composition of LCFA, rather than reducing energy charge, induces the activation of AMPK in VSMC. In fact, Watt et al previously showed that palmitate treatment induces AMPK activation in L6 skeletal muscle myotubes in the absence of detectable changes in free AMP and glycogen content,^47^ although precise molecular mechanisms whereby palmitate induces AMPK have not been described.

Our study newly identifies ROS as an upstream effector that induces AMPK activation in response to increased palmitate availability. We previously showed that dysregulation of Elovl6‐mediated LCFA metabolism induces ROS production and pulmonary fibrosis.^13^ Similar findings were observed in the present study, implying that ROS production in Elovl6‐deficient cells is not cell type specific. We now demonstrate several lines of evidence indicating the causative role of ROS production for AMPK/KLF4 activation in Elovl6‐deficient VSMC: (1) ROS production was increased in Elovl6^−/−^ mice and siElovl6‐transfected VSMC; (2) palmitate induced and oleate reduced ROS production; and (3) exposure of VSMC to H_2_O_2_ recapitulated the findings of Elovl6‐deficient VSMC, including an increased phosphorylation of AMPK, elevation of KLF expression, and reduced VSMC gene expression. These data support the accumulating evidence indicating that increased ROS production in response to saturated LCFA overload plays a critical role in the regulation of cellular function.^48^, ^49^, ^50^, ^51^ Then, what are the mechanisms by which ROS induces AMPK activation? Although this question remains to be addressed, we recently found that the expression of Sestrin 1 and 2, which function as antioxidants or as AMPK activators in response to metabolic stress,^52^ was increased in HASMC with Elovl6 knockdown. Future work should be warranted to identify the mechanism of ROS generation and ROS‐induced AMPK activation in Elovl6‐deficient VSMC.

Our results have potential clinical implications, especially related to the vascular complications in type 2 diabetes and obese subjects because previous studies showed that Elovl6 expression is transcriptionally induced by insulin and overnutrition through the action of the lipogenic transcription factor, SREBP1c^9^, ^53^ in hepatocytes. We found that PDGF‐BB and hypoxia efficiently increased Elovl6 gene expression in HASMC (Figures S3 and S10). Thus, it is tempting to speculate that overnutrition‐induced elevation of Elovl6 expression in VSMC may contribute to the development of advanced atherosclerotic lesions and neointima by priming the cells for enhanced mitogenic response.

Our findings that mTOR and p21 expression was profoundly affected by Elovl6 knockdown seem to support the concept that growth and cell division are coordinately regulated to ensure that cells keep a fairly constant size.^54^ Our study further highlights the central role of AMPK not only as a sensor for AMP/ATP levels but also as a sensor of metabolic stress caused by change in lipid composition. There has been a general consensus that AMPK activation directly and indirectly (via TSC2 and Raptor) suppresses mTOR activity to limit protein synthesis. Accordingly, our data corroborate the notion that AMPK is a regulator of cellular proliferation and protein synthesis by coordinating multiple cellular processes to maintain energy homeostasis and normal cellular function.

It is possible that activation of AMPK is expected to promote preferential partitioning of intracellular fatty acids away from esterification toward oxidation. Thus, activation of AMPK could be an endogenous protective mechanism to ameliorate lipotoxic effects of palmitate leading to endoplasmatic reticulum (ER) stress, inflammation, and insulin resistance^55^, ^56^, ^57^ in Elovl6‐deficient VSMC. Further studies are needed to verify our hypothesis that activation of AMPK/KLF4 signaling by loss of Elovl6 is protective against atherosclerosis by using double knockout mice of Elovl6 and apoE.

In summary, our study reveals a previously unrecognized role of Elovl6‐driven LCFA metabolism as an intracellular cue for the regulation of VSMC phenotypic switching. Furthermore, we provide evidence for the role of LCFA composition and ROS production in the activation of AMPK/KLF4 signaling in VSMC. These findings indicate that strategies aimed at Elovl6‐driven LCFA metabolism and AMPK/KLF4 signaling may represent a successful approach to limit atherosclerosis and postangioplasty restenosis.

## Sources of Funding

This work was supported by a Grant‐in‐Aid for Scientific Research from the Japan Society for the Promotion of Science (to H.Sunaga, Matsui, Iso, Yokoyama, and Kurabayashi), Gunma University Initiative for Advanced Research (to Kurabayashi), and Japan Heart Foundation and Astellas Grant for Research on Atherosclerosis Update (to H.Sunaga, Matsui).

## Disclosures

None.

## Supporting information


**Data S1.** Supplemental Experimental Procedures
**Figure S1.** Expression of Elovl6 in intimal thickening lesions of the human coronary artery.
**Figure S2.** Adenovirus‐mediated overexpression of Elovl6 in HASMC.
**Figure S3.** Induction of Elovl6 expression by PDGF‐BB in HASMC.
**Figure S4.** Overexpression of Elovl6‐modulated cell cycle regulators in HASMC.
**Figure S5.** Elovl6 overexpression alters the fatty acid composition in HASMC.
**Figure S6.** Changes of Elovl6 expression in HASMC affect KLF4 expression.
**Figure S7.** Depletion of KLF4 affects cell cycle regulators and proliferation in HASMC with or without Elovl6 knockdown.
**Figure S8.** KLF4 knockdown affects SMC marker gene expression in a context‐dependent manner.
**Figure S9.** KLF4 knockdown has no effects on phosphorylation of AMPK.
**Figure S10.** Induction of Elovl6 expression by hypoxic stress in HASMC.
**Table S1.** Human Primer Sequences Used for qRT‐PCR
**Table S2.** Mouse Primer Sequences Used for qRT‐PCR
