# Supplementary material for: Elongation of Long‐Chain Fatty Acid Family Member 6 (Elovl6)‐Driven Fatty Acid Metabolism Regulates Vascular Smooth Muscle Cell Phenotype Through AMP‐Activated Protein Kinase/Krüppel‐Like Factor 4 (AMPK/KLF4) Signaling
Source: J Am Heart Assoc. 2016 Nov 23;5(12):e004014. doi: 10.1161/JAHA.116.004014 (PMC5210431; doi:10.1161/JAHA.116.004014)
Supplement: Supplementary file 1 — Data S1. Supplemental Experimental Procedures Figure S1. Expression of Elovl6 in intimal thickening lesions of the human coronary artery. Figure S2. Adenovirus‐mediated overexpression of Elovl6 in HASMC. Figure S3. Induction of Elovl6 expression by PDGF‐BB in HASMC. Figure S4. Overexpression of Elovl6‐modulated cell cycle regulators in HASMC. Figure S5. Elovl6 overexpression alters the fatty acid composition in HASMC. Figure S6. Changes of Elovl6 expression in HASMC affect KLF4 expression. Figure S7. Depletion of KLF4 affects cell cycle regulators and proliferation in HASMC with or without Elovl6 knockdown. Figure S8. KLF4 knockdown affects SMC marker gene expression in a context‐dependent manner. Figure S9. KLF4 knockdown has no effects on phosphorylation of AMPK. Figure S10. Induction of Elovl6 expression by hypoxic stress in HASMC. Table S1. Human Primer Sequences Used for qRT‐PCR Table S2. Mouse Primer Sequences Used for qRT‐PCR [file JAH3-5-e004014-s001.pdf]

SUPPLEMENTAL MATERIAL

Data S1. Supplemental Experimental Procedures

Animals

C57BL/6 strain (wild-type, WT) mice and Wistar rats were purchased from CLEA Japan Inc.. *Elovl6*$^{-/-}$ mice were kindly gifted from Dr. H. Shimano (Tsukuba University). These mice were intercrossed into *Elovl6*$^{-/-}$ or *Elovl6*$^{-/+}$ mice. Littermates were genotyped by PCR.

Human arterial tissue

Eight human coronary artery sections, containing diffuse intimal thickening or apparently normal pathology, were obtained from patients with ischemic or non-ischemic heart disease.

Tissue preparation and immunohistochemistry

Following the induction of deep anesthesia, mice were killed and perfused via the left ventricle with phosphate buffered saline (PBS), followed by perfusion fixation with 10% formalin for 10 min. The right femoral artery was carefully harvested and embedded in paraffin. Human arterial tissues were obtained from patients at autopsy and tissue samples were fixed in formalin and embedded in paraffin. Each serial section (4-μm) was prepared for the histological analysis.

Sections were deparaffinized in xylene and rehydrated prior to antigen retrieval by boiling in citrate buffer. Elovl6 (ABGENT, AP6524a, 1:50), SMα-actin (Dako corp., M0851, 1:500), and Nitrotyrosine (Cell Signaling Technology, #9691, 1:50) staining was performed using the Vectastain Elite ABC kit (Vector Laboratories) according to the manufacturer's instruction, with 3,3'-diaminobenzidine (DAB) as the chromogenic substrate. Sections were stained with hematoxylin and eosin (H&E) and elastica van Gieson (EVG) using standard procedures. The immunohistochemistry of artery cross-sections was examined by an image analysis

(WinROOF, Mitani Shoji Co.). In each artery, measurements of the intimal (I, tissue between the lumen and internal elastic lamina) and medial (M, tissue between the internal and external elastic lamina) areas were obtained, and the intimal/media area ratio (I/M ratio) was calculated[1-3]. Ki-67 (Thermo Scientific, SP6, 1:200) -positive cells were calculated as the number of cells that co-localized with 4', 6-diamidino-2-phenylindole (DAPI, Dojindo, 1:1000) in the neointimal thickening area of the femoral artery in mice.

Balloon injury to the rat carotid artery

The carotid artery of rats was injured with a balloon as described previously[4]. In brief, 8-week-old male Wister (n=12) rats weighing approximately 300 g were injured with a 2F balloon catheter (Edwards Lifesciences). The animals were euthanized 14 days after the balloon injury and the thoracic aorta was harvested.

Cell culture

Human aortic smooth muscle cells (HASMC) were purchased from Kurabo Ltd. HASMC were cultured at 37°C in a 5% CO_2 atmosphere in Humedia-SB2 medium supplemented with 5% fetal bovine serum (FBS), 0.5 μg/ml of recombinant human epidermal growth factor (EGF), 2 μg/ml of recombinant human basic fibroblast growth factor (bFGF), 5 mg/ml of insulin, 50 mg/ml of gentamycin, and 50 μg/ml of amphotericin B. HASMC were used between passages 4 and 8 in these experiments. Serum-starved HASMC with 0.1% BSA (24 hours) were incubated in the presence or absence of BSA-conjugated palmitate or oleate (Sigma, 250 μM). Serum-starved HASMC were also treated with 1 mM AICAR (Toronto Research Chemicals) or 10 μM Compound C (Merck). Cells were also treated with vehicle as control, TGF-β （Roche） or PDGF-BB (R＆D systems). HASMC was treated with 100 μM H_2O_2 (Wako) in the presence or absence of 1 unit catalase (Merck Millipore).

RNA isolation and quantitative real-time reverse transcription (qRT-PCR)

Total RNA was extracted from the mouse aorta and cultured HASMC using ISOGEN regent (Takara Bio) according to the manufacturer's protocol. One microgram of RNA was used for reverse transcription with the RNA LA PCR Kit (Takara Bio) and qRT-PCR analysis was performed using the THUNDERBIRD SYBR qPCR Mix (TOYOBO) according to the manufacturers' protocols. QPCR was carried out using a MX3000P quantitative system (Stratagene). All primer sequences are shown in Supplemental Table I and Table II. Each experiment used three samples and was performed three times.

Construction of siRNA oligonucleotides and transfection

siRNA oligonucleotides (human siElovl6: CUU UUG AAC AGA AGA GUA AAU, human sip21: CGA CUG UGA UGC GCU AAU G, CCU AAU CCG CCC ACA GGA A, CGU CAG AAC CCA UGC GGC A, AGA CCA GCA UGA CAG AUU U, human siKLF4: CCA GAG GAG CCC AAG CCA ATT, siGFP: GUU CAG CGU GUC CGG CGA GTT) were purchased from BONAC Corporation and transfected with Lipofectamine RNAiMAX Regent (Invitrogen) according to the manufacturer's protocol. siGFP concentration used was 50 nM, which has little, if any, off-target effects[5]. In order to effectively inhibit the four transcript variants of human p21 expression, we used four sequences of p21 siRNA that were mixed in equal amounts.

[^{3}H]-thymidine uptake

The uptake of radiolabeled [^{3}H]-thymidine was assayed as described previously[6], with some modifications. Briefly, HASMC were seeded on 24-well plates (2.5×10^4 cells/well) and cultured for 24 hours in Hu-media containing 5% FBS. These cells were treated with each adenovirus or siRNA followed by serum-starvation (0.5% FBS) for 24 hours. Serum-starved HASMC with 0.1% BSA (24 hours) were also treated with BSA-

conjugated palmitate or oleate (250 μM each) for 24 hours. The cells were incubated with [^{3}H]-thymidine (1 mCi/well; Perkin Elmer) in a 5% CO_2 atmosphere at 37°C. After a 5 hours incubation, the cells were washed with ice-cold PBS and the reaction was stopped by the addition of ice-cold 5% trichloroacetic acid (TCA) for 30 min. The cells were digested in 0.5 M NaOH for 1 hour and aliquots were used to determine the radioactive count with a scintillator counter (Aloca LSC3000).

Scratch wound healing assay

The measurement of migration by the scratch mobility assay was performed as described previously[7]. Briefly, HASMC transduced with the adenovirus or siRNA were seeded on 6-well plates. After 24 hours, a single uniform scratch was made on the monolayer in each well using a sterile 200-μl pipette tip. The monolayer was rinsed three times and placed in Hu-media with 0.5% FBS. Phase contrast images were captured immediately and after 8 hours or 24 hours incubation, and a digital image of the scar was taken at a magnification of ×40. Each experiment was performed in duplicate and three independent assays were performed (n=6).

Boyden chamber assay

Boyden chamber assay was performed as previously described[7]. Briefly, HASMC which were transfected with siElovl6 or siGFP for 48 h were plated (2.5×10^4/well) in the upper chamber of transwells (6.5-mm diameter, 5.0-μm pore size polycarbonate membrane, Neuro Probe). The cells were returned to the 37°C 5% CO_2 incubator for 4 h, rinsed with PBS, and cells remaining at the top of the polycarbonate membrane were removed. The cells that had migrated through pores to the lower surface were fixed in methanol, stained with a Diff-Quick kit (Sysmex). Membranes were mounted on microslides and four random microscopic fields per well were quantified. Each experiment was conducted in duplicate and three separate experiments were performed (n=6-

7).

Fatty acid composition

Lipids from the mouse aorta and HASMC were extracted using the Bligh and Dyer method as described previously[8]. Briefly, arterial tissues and cells were extracted with chloroform/methanol (1:2, v/v) solution. In order to break monophase, 1 M NaCl solution and chloroform were then added, and incubated on ice for 10 min. After centrifugation at 2,000 rpm for 5 min, the aqueous solution was discarded and the chloroform phase was transferred to a test tube. The chloroform phase was evaporated using nitrogen gas, and the lipids obtained were subjected to a fatty acid composition analysis at an external laboratory (SRL Inc.).

Western blot analysis

Tissue samples and cells were homogenized on ice in RIPA buffer (20 mM Tris-HCl [pH 7.4], 150 mM NaCl, 1% NP-40, 1% sodium deoxycholate, 0.1% SDS, and containing complete mini and phosSTOP solution (Roche)). The mixture was centrifuged at 15,000 rpm for 30 min and the supernatant was subjected to SDS-PAGE. Protein concentrations were determined by the Bradford method using a colorimetric assay (Bio-Rad). Western blot analysis was performed according to standard procedures using the following primary antibodies: rabbit monoclonal phospho-AMPK (Cell Signaling Technology, #2535, 1:250), AMPK (Cell Signaling Technology, #2603, 1:500), ACC (Cell Signaling Technology, #3676, 1:500), phospho-mTOR (Cell Signaling Technology, #5536, 1:250), mTOR (Cell Signaling Technology, #2983, 1:500), p21 (Cell Signaling Technology, #2947, 1:500), KLF4 (Cell Signaling Technology, #4038, 1:250), β-actin (Cell Signaling Technology, #4970, 1:500), and rabbit polyclonal phospho-ACC (Cell Signaling Technology, #3661, 1:250), phospho-p53 (Cell Signaling Technology, #9284, 1:250), SMα-actin (Dako corp., M0851, 1:100), SM22α (Abcam, ab14106,

1:1000) and mouse polyclonal p53 (Santa Cruz Biotechnology, #Sc-126, 1:100). Antigens were revealed by Immobilon Western HRP Substrate (Millipore) after an incubation with horseradish peroxidase-conjugated anti-rabbit or mouse IgG. The density of a band was quantified using ImageJ software.

Preparation of fatty acid solution

Palmitate was conjugated to fatty acid-free bovine serum albumin at a 3.5:1 molar ratio by dissolving them in ethanol and mixing with an aqueous BSA solution (BSA in phosphate-buffered saline, PBS) at 37°C until homogeneous. They were then passed through a 0.2-μM filter.

Estimation of ROS generation

Intracellular ROS levels were detected with the oxidant-sensitive fluorogenic probes 5-(and 6)-chloromethyl-2',7'-dichlorodihydrofluorescein diacetate, acetyl ester (CM-H_2DCFDA, Invitrogen). siRNA-transfected HASMC was incubated with 10 μM CM-H_2DCFDA in serum-free medium for 30 min at 37°C. Otherwise, the cells were incubated in the presence or absence of palmitic acid and oleic acid for 15 min after 30 min incubation of 10 μM CM-H_2DCFDA. After incubation at 37°C, fluorescence was detected.

Ethical approval

Human coronary artery tissues with atherosclerosis were obtained from patients at autopsy with written informed consent of their family at Gunma University Hospital. This protocol was approved by the Institutional Review Board at the Gunma University Hospital.

Figure S1. Expression of Elovl6 in intimal thickening lesions of the human coronary artery.

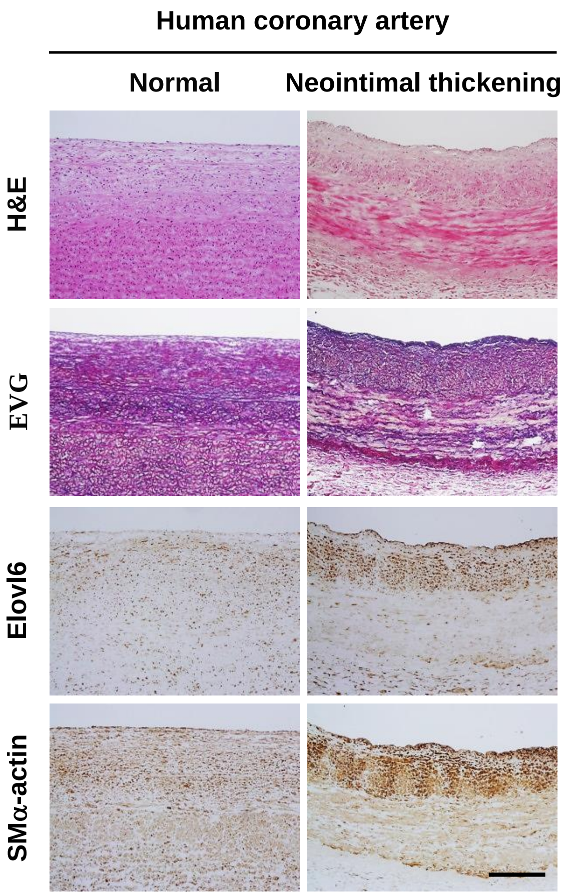

Eight human coronary artery sections, containing diffuse intimal thickening or apparently normal pathology, from a patient with ischemic or non-ischemic heart disease were stained with H&E, EVG, and with antibodies against Elovl6 and SMα-actin. The expression of Elovl6 was co-localized with SMα-actin in the thin medial layer in a normal coronary artery, and increased in intimal thickening lesions of human coronary artery. Scale bar = 200 μm. H&E, hematoxylin-eosin; EVG, elastica van Gieson; Elovl6, elongation of long-chain fatty acid family member 6; SMα-actin, smooth muscle alpha-actin.

Figure S2. Adenovirus-mediated overexpression of Elovl6 in HASMC.

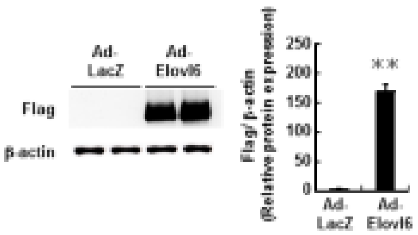

Western blot analysis with an anti-flag M2 antibody in HASMC transduced with Ad-Elovl6 (n=6) or Ad-LacZ (n=6). Flag-tagged Elovl6 was markedly up-regulated in HASMC infected with Ad-Elovl6. β-actin was measured as an internal control. This experiment was performed at least three times. Values are represented as the means ± SEM of three experiments. *$p<0.05$, **$p<0.01$, as measured by the Mann-Whitney U-test. HASMC, human aortic smooth muscle cells; Ad-LacZ, adenoviral vector encoding lacZ gene; Ad-Elovl6, adenoviral vector encoding elongation of long-chain fatty acid family member 6 gene.

Figure S3. Induction of Elovl6 expression by PDGF-BB in HASMC.

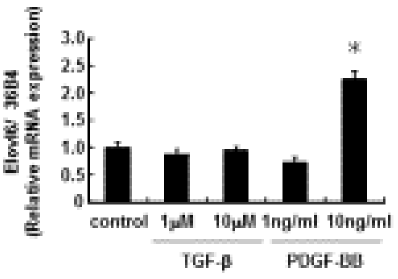

qRT-PCR for Elovl6 mRNA in HASMC. PDGF-BB induced Elovl6 mRNA levels in a dose-dependent manner (control, TGF-β, PDGF-BB: n=6). 36B4 mRNA levels were measured as internal controls. This experiment was performed at least three times. Values are represented as the means ± SEM of three experiments. *$p<0.05$, as measured by the Tukey-Kramer test. Elovl6, elongation of long-chain fatty acid family member 6; HASMC, human aortic smooth muscle cells; PDGF-BB, platelet-derived growth factor-BB, TGF-β, transforming growth factor-beta.

Figure S4. Overexpression of Elovl6 modulated cell cycle regulators in HASMC.

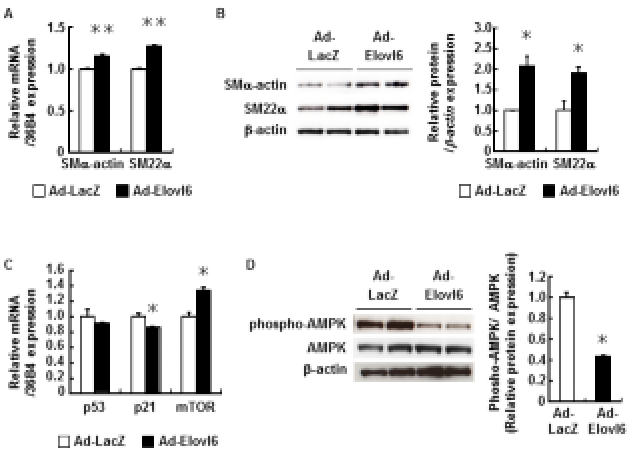

(A, B) qRT-PCR for SMα-actin and SM22α mRNA levels (A) and western blot assay for protein levels (B) in HASMC infected with Ad-Elovl6 (n=6) or Ad-LacZ (n=5) for 48 hours. Overexpression of Elovl6 increased the SM22α and SMα-actin mRNA and protein levels in HASMC. (C) qRT-PCR for the expression of p53, p21 and mTOR mRNA in HASMC infected with Ad-Elovl6 (n=6) or Ad-LacZ (n=7) for 48 hours. p21 mRNA levels were decreased while mTOR mRNA levels were increased by Elovl6 overexpression. (D) Western blot analysis of AMPK in HASMC infected with Ad-Elovl6 (n=6) or Ad-LacZ (n=6) for 48 hours. Overexpression of Elovl6 suppressed phosphorylation of AMPK. 36B4 for qRT-PCR and β-actin for western blot were measured as an internal control. Western blot data for the phosphorylation of AMPK were normalized by total AMPK in the

same samples, and expressed as a fold increase from the mean level of control group. Each experiment was performed at least three times. All values are represented as the means ± SEM of three experiments. *$p<0.05$, **$p<0.01$, as measured by the Mann-Whitney U-test. SMα-actin, smooth muscle alpha-actin; SM22α, smooth muscle protein 22-alpha; HASMC, human aortic smooth muscle cells; Ad-LacZ, adenoviral vector encoding lacZ gene; Ad-Elovl6, adenoviral vector encoding elongation of long-chain fatty acid family member 6 gene; mTOR, mammalian target of rapamycin; AMPK, AMP-activated protein kinase.

Figure S5. Elovl6-overexpression altered the fatty acid composition in HASMC.

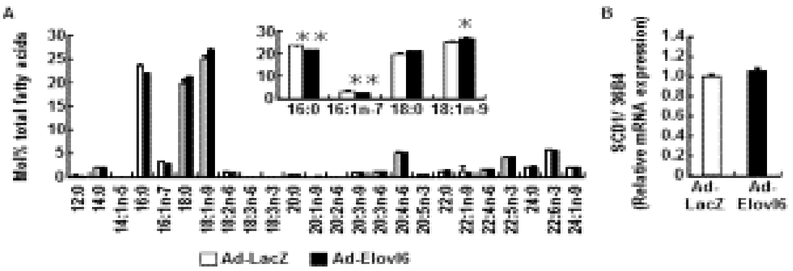

(A) Fatty acid composition of the neutral lipid extract from HASMC infected with Ad-Elovl6 (n=5) or Ad-LacZ (n=4) for 48 hours. The adenovirus-mediated overexpression of Elovl6 decreased the relative amounts of palmitate (C16:0) and palmitoleate (C16:1 n-7), and increased those of oleate (C18:1 n-9) more than the infection with Ad-LacZ. (B) qRT-PCR for SCD1 mRNA in HASMC infected with Ad-Elovl6 (n=6) or Ad-LacZ (n=5). The overexpression of Elovl6 did not change SCD1 mRNA levels. 36B4 was measured as an internal control. mRNA levels in the Ad-LacZ control group were normalized to a value of 1, while those in Ad-Elovl6 cells are shown relative to the control level. Each experiment was performed at least three times. All values are

represented as the means ± SEM of three experiments. *$p<0.05$, **$p<0.01$, as measured by the Mann-Whitney U-test. HASMC, human aortic smooth muscle cells; Ad-LacZ, adenoviral vector encoding lacZ gene; Ad-Elovl6, adenoviral vector encoding elongation of long-chain fatty acid family member 6 gene; SCD1, stearoyl-CoA desaturase 1.

Figure S6. Changes of Elovl6 expression in HASMC affect KLF4 expression.

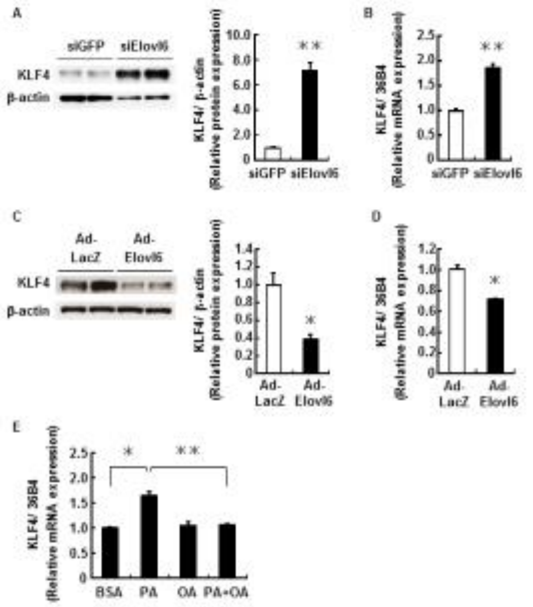

(A) Western blot analysis of KLF4 expression in HASMC transfected with Elovl6 (n=6) or GFP siRNA (n=6). KLF4 protein levels were significantly increased by the siRNA-mediated knockdown of Elovl6. (B) qRT-PCR analysis for KLF4 mRNA in HASMC with siRNA-mediated knockdown of Elovl6 (siGFP: n=7 ; siElovl6 : n=6). (C) Western blot analysis of KLF4 in HASMC infected with Ad-Elovl6 (n=6) or Ad-LacZ (n=6) for 48 hours. Overexpression of Elovl6 significantly decreased KLF4 protein levels. (D) qRT-PCR for KLF4 mRNA in HASMC infected with Ad-Elovl6 (n=6) or Ad-LacZ (n=6) for 48 hours. KLF4 mRNA levels were decreased by Elovl6 overexpression. (E) qRT-PCR for KLF4 mRNA in HASMC treated with fatty acids. The PA (C16:0) treatment significantly increased the KLF4 mRNA levels, whereas the OA (C18:1n-9) treatment inhibited this induction (BSA: n=8; PA, OA, PA + OA: n=7). 36B4 for qRT-PCR and β-actin for western blot were measured

as an internal control. Each experiment was performed at least three times. All values are represented as the means ± SEM of three experiments. *$p<0.05$, **$p<0.01$, as measured by the Mann-Whitney U-test (A-D) or Tukey-Kramer test (E). KLF4, krüppel-like factor 4; HASMC, human aortic smooth muscle cells; Ad-LacZ, adenoviral vector encoding lacZ gene; Ad-Elovl6, adenoviral vector encoding elongation of long-chain fatty acid family member 6 gene; PA, palmitic acid; OA, oleic acid; BSA, fatty acid-free bovine serum albumin.

Figure S7. Depletion of KLF4 affects cell cycle regulators and proliferation in HASMC with or without Elovl6 knockdown.

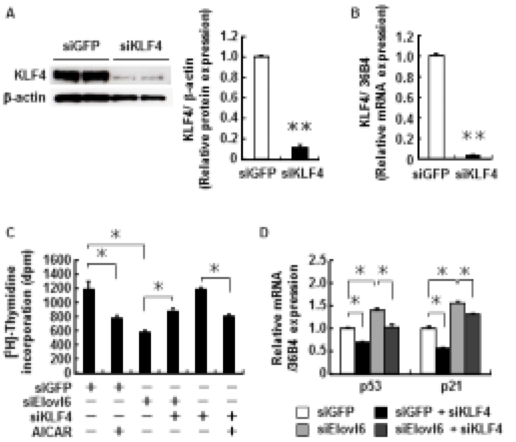

(A, B) Western blot analysis (A) or qRT-PCR (B) of KLF4 expression in HASMC transfected with KLF4 (n=6) or GFP siRNA (n=5). KLF4 expression was significantly decreased by the siRNA-mediated knockdown of KLF4. (C) [^{3}H]-thymidine uptake assay in HASMC transfected with Elovl6, KLF4 or GFP siRNA in the presence or absence of 1mM AICAR. Concomitant treatment of siKLF4 and siElovl6 partially reversed siElovl6-triggered inhibition of VSMC proliferation (siGFP, siElovl6, siKLF4: n=6; siElovl6 + siKLF4: n=7; AICAR: n=5). (D) Effects of KLF4 gene silencing on Elovl6 siRNA-induced cell cycle regulatory proteins in HASMC by qRT-PCR. Elovl6 depletion-induced expression of p53 and p21 was cancelled by the siRNA-

mediated knockdown of KLF4 (siGFP, siElovl6: n=6; siGFP or siElovl6 + siKLF4: n=7). Each experiment was performed at least three times. All values are represented as the means ± SEM of three experiments. *$p<0.05$, **$p<0.01$, as measured by the Mann-Whitney U-test (A, B) or Tukey-Kramer test (C, D). KLF4, krüppel-like factor 4; HASMC, human aortic smooth muscle cells; AICAR, 5-aminoimidazole-4-carboxamide ribonucleotide; Elovl6, elongation of long-chain fatty acid family member 6; VSMC, vascular smooth muscle cells.

Figure S8. KLF4 knockdown affects SMC marker gene expression in a context-depend manner.

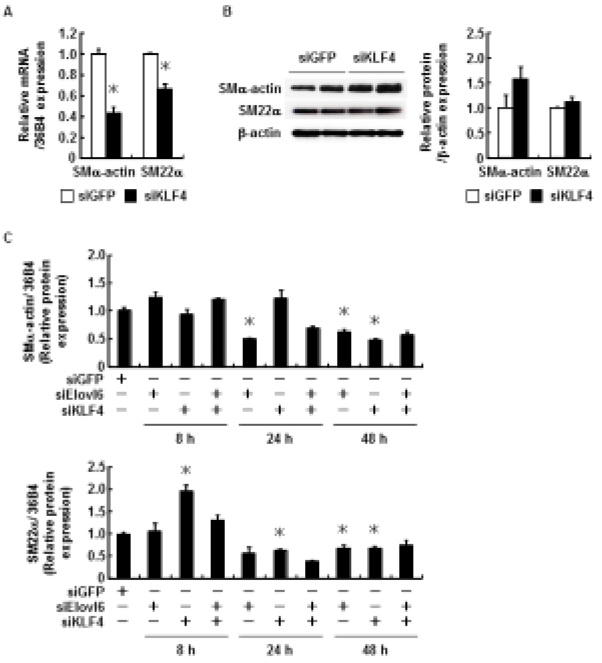

(A, B) qRT-PCR for SMα-actin and SM22α mRNA (A) and western blot assay for protein (B) in HASMC transfected with KLF4. SMα-actin and SM22α mRNA levels were significantly decreased, but protein levels were not changed by siRNA-mediated knockdown of KLF4 (siGFP: n=7; siKLF4: n=8). 36B4 for qRT-PCR and β-actin for western blot were measured as an internal control. (C) qRT-PCR for the time-course of SMα-actin or SM22α mRNA levels, normalized by mRNA levels of 36B4, in HASMC transfected with siRNA targeting Elovl6 or KLF4. siRNA-mediated Elovl6 or KLF4 knockdown increased SMα-actin or SM22α expressions at an early time (8-24h), however, Elovl6 or KLF4 knockdown markedly suppressed SMC marker gene expressions 48 h after transfection (siGFP, siElovl6, siKLF4 : n=7 ; siElovl6 + siKLF4 : n=7). Each

experiment was performed at least three times. All values are represented as the means ± SEM of three experiments. *$p<0.05$, **$p<0.01$, as measured by the Mann-Whitney U-test (A, B) or Tukey-Kramer test (C). SMα-actin, smooth muscle alpha-actin; SM22α, smooth muscle protein 22-alpha; HASMC, human aortic smooth muscle cells; KLF4, krüppel-like factor 4; Elovl6, elongation of long-chain fatty acid family member 6; SMC, smooth muscle cell.

Figure S9. KLF4 knockdown had no effects on phosphorylation of AMPK.

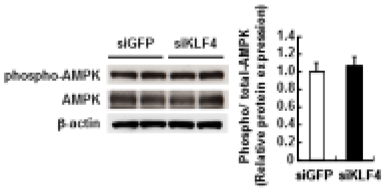

Western blot analysis of AMPK phosphorylation in HASMC transfected with KLF4 (n=6) or GFP siRNA (n=5). Phosphorylation of AMPK was not changed by siRNA-mediated knockdown of KLF4. β-actin was measured as an internal control. This experiment was performed at least three times. All values are represented as the means ± SEM of three experiments, measured by the Mann-Whitney U-test. AMPK, AMP-activated protein kinase; HASMC, human aortic smooth muscle cells; KLF4, krüppel-like factor 4.

Figure S10. Induction of Elovl6 expression by hypoxic stress in HASMC.

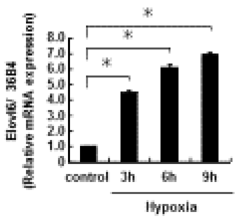

qRT-PCR for Elovl6 mRNA in HASMC. Hypoxic stress markedly induced Elovl6 mRNA levels, normalized by mRNA levels of 36B4, in a time-dependent manner (control, hypoxia: n=6). This experiment was performed at least three times. Values are represented as the means ± SEM of three experiments. *$p<0.05$, as measured by the Tukey-Kramer test. Elovl6, elongation of long-chain fatty acid family member 6; HASMC, human aortic smooth muscle cells.

Table S1. Human primer sequences used for qRT-PCR

Target (Human)	Sense	Anti-sense
Elovl6	5’-CGAGAATGAAGCCATCCAAT-3’	5’-CCCGCAAGGCATAGTAAGAG-3
SCD1	5’-CCACCGCTCTTACAAAGCTC-3’	5’-TAGTTGTGGAAGCCCTCACC-3
SMα-actin	5’-ATGAGGGCTATGCCTTGCCC-3’	5’-CCCGATGAAGGATGGCTGGA-3’
SM22α	5’-AACAGCCTGTACCCTGATGG-3’	5’-ATGACATGCTTTCCCTCCTG-3’
p53	5’-GTGGAAGGAAATTTGCGTGT-3’	5’-TCTGAGTCAGGCCCTTCTGT-3
p21	5’-GAGCGATGGAACTTCGACTT-3	5’-GGCGTTTGGAGTGGTAGAAA-3
mTOR	5’-AGTGGACCAGTGGAAACAGG-3	5’-TCCGGCTGCTGCTGTAGCTTATT-3
KLF4	5’-ACCCTGGGTCTTGAGGAAGT-3	5’-ATGTGTAAGGCGAGGTGGTC-3
36B4	5’-ATCCCTGACGACGCACCGCCGTGA-3	5’-TGCATCTGCTTGGAGCCCACGTT-3

Table S2. Mouse primer sequences used for qRT-PCR

Target (Mouse)	Sense	Anti-sense
Elovl6	5’-CCCGAACTAGGTGACACGAT-3’	5’-TACTCAGCCTTCGTGGCTTT-3’
SCD1	5’-GCTGGGCAGGAACTAGTGAG-3’	5’-GGTAGGGAGGATCTGGAAGC-3’
SMα-actin	5’-AGACAGCTATGTGGGGGATG-3’	5’-GAAGGAATAGCCACGCTCAG-3’
SM22α	5’-TCCAGTCCACAAACGACCAAGC-3’	5’-GAATTGAGCCACCTGTTCCATCTG-3’
p53	5’-ATCTGGACGACAGGCACACT-3’	5’-CTTCGGGTAGCTGGAGTGAG-3’
p21	5’-GTCCAATCCTGGTGATGTCC-3’	5’-CAGGGCAGAGGAAGTACTGG-3’
mTOR	5’-CATCCCCAAGGTGCTACAGT-3’	5’-CAAACCACAGGGTGAGGACT-3’
KLF4	5’-CAGCTTCATCCTCGTCTTCC-3’	5’-CGGGACTCAGTGTAGGGGTA-3’
36B4	5’-ATCCCTGACGACGCACCGCCGTGA-3	5’-TGCATCTGCTTGGAGCCCACGTT-3

Supplemental References:

1. Gallo R, Padurean A, Toschi V, Bichler J, Fallon JT, Chesebro JH, Fuster V, Badimon JJ. Prolonged thrombin inhibition reduces restenosis after balloon angioplasty in porcine coronary arteries. *Circulation*. 1998; 97:581-588.
2. Lin RY, Reis ED, Dore AT, Lu M, Ghodsi N, Fallon JT, Fisher EA, Vlassara H. Lowering of dietary advanced glycation endproducts (AGE) reduces neointimal formation after arterial injury in genetically hypercholesterolemic mice. *Atherosclerosis*. 2002; 163:303-311.
3. Roque M, Fallon JT, Badimon JJ, Zhang WX, Taubman MB, Reis ED. Mouse model of femoral artery denudation injury associated with the rapid accumulation of adhesion molecules on the luminal surface and recruitment of neutrophils. *Arterioscler Thromb Vasc Biol*. 2000; 20:335-342.
4. Doi H, Iso T, Yamazaki M, Akiyama H, Kanai H, Sato H, Kawai-Kowase K, Tanaka T, Maeno T, Okamoto E, Arai M, Kedes L, Kurabayashi M. HERP1 inhibits myocardin-induced vascular smooth muscle cell differentiation by interfering with SRF binding to CArG box. *Arterioscler Thromb Vasc Biol*. 2005; 25:2328-2334.
5. Tschuch C, Schulz A, Pscherer A, Werft W, Benner A, Hotz-Wagenblatt A, Barrionuevo LS, Lichter P, Mertens D. Off-target effects of siRNA specific for GFP. *BMC Mol Biol*. 2008; 9:60.
6. Farhat MY, Vargas R, Dingaan B, Ramwell PW. In vitro effect of oestradiol on thymidine uptake in pulmonary vascular smooth muscle cell: role of the endothelium. *Br J Pharmacol*. 1992; 107:679-683.
7. Aoyagi-Ikeda K, Maeno T, Matsui H, Ueno M, Hara K, Aoki Y, Aoki F, Shimizu T, Doi H, Kawai-Kowase K, Iso T, Suga T, Arai M, Kurabayashi M. Notch induces myofibroblast differentiation of alveolar epithelial cells via transforming growth factor-{beta}-Smad3 pathway. *Am J Respir Cell Mol*

Biol. 2011; 45:136-144.

8. Bligh EG, Dyer WJ. A rapid method of total lipid extraction and purification. *Can J Biochem Physiol*. 1959; 37:911-917.
